# Publisher Correction: Mesoporous multimetallic nanospheres with exposed highly entropic alloy sites

**DOI:** 10.1038/s41467-023-40653-8

**Published:** 2023-08-11

**Authors:** Yunqing Kang, Ovidiu Cretu, Jun Kikkawa, Koji Kimoto, Hiroki Nara, Asep Sugih Nugraha, Hiroki Kawamoto, Miharu Eguchi, Ting Liao, Ziqi Sun, Toru Asahi, Yusuke Yamauchi

**Affiliations:** 1 Research Center for Materials Nanoarchitectonics and Research Center for Advanced Measurement and Characterization, National Institute for Materials Science (NIMS), 1-1 Namiki, Tsukuba, Ibaraki 305-0044 Japan; 2 Faculty of Science and Engineering, Waseda University, 3-4-1 Okubo, Shinjuku, Tokyo 169-8555 Japan; 3 Australian Institute for Bioengineering and Nanotechnology (AIBN) and School of Chemical Engineering, The University of Queensland, Brisbane, QLD 4072 Australia; 4 Hitachi High-Tech Corporation, 882, Ichige, Hitachinaka-shi, Ibaraki 312-0033 Japan; 5 School of Mechanical, Medical and Process Engineering, Queensland University of Technology, Brisbane, QLD 4001 Australia; 6 School of Chemistry and Physics, Queensland University of Technology, Brisbane, QLD 4001 Australia; 7 Department of Materials Process Engineering, Graduate School of Engineering, Nagoya University, Nagoya, 464–8603 Japan

**Keywords:** Materials chemistry, Nanoscale materials, Metals and alloys

Correction to: *Nature Communications* 10.1038/s41467-023-39157-2, published online 13 July 2023

The original version of the Supplementary Information associated with this Article included incorrect Supplementary Datasets. Dataset 1 reported an editorial policy checklist; Dataset 3 reported a general description of Supplementary Figures and Tables contained in the Supplementary Information PDF file; Dataset 4 contained editorial instructions to the authors for the preparation of the final version of the article. Datasets 1, 3, and 4 have been removed in the corrected version of the Article.

In addition, the Reporting Summary and the Source Data file were originally published with the wrong titles of Dataset 2 and Dataset 5. The HTML version of the article has been corrected.

### Supplementary information

Reporting Summary

### Source data

Source Data

