# Supplementary material for: Mesoporous multimetallic nanospheres with exposed highly entropic alloy sites
Source: Nat Commun. 2023 Jul 13;14:4182. doi: 10.1038/s41467-023-39157-2 (PMC10344865; doi:10.1038/s41467-023-39157-2)
Supplement: Supplementary file 1 — Supplementary Information [file 41467_2023_39157_MOESM1_ESM.pdf]

Supplementary Information

Mesoporous multimetallic nanospheres with exposed highly entropic alloy sites

Yunqing Kang, Ovidiu Cretu, Jun Kikkawa, Koji Kimoto, Hiroki Nara, Asep Sugih Nugraha, Hiroki Kawamoto, Miharu Eguchi, Ting Liao,* Ziqi Sun, Toru Asahi, and Yusuke Yamauchi*

CONTENTS

Supplementary Figures

Supplementary Figure 1. The photographs of micellization process through the Tyndall effect and a chemical reduction process of PtPdRhRuCu MMNs.

Supplementary Figure 2. FTIR spectra of bare PEO_{10500}-*b*-$PMMA_{18000}$, PtPdRhRuCu MMNs before and after solvent extraction.

Supplementary Figure 3. SEM images and corresponding particle/pore size distribution of PtPdRhRuCu MMNs.

Supplementary Figure 4. The SAXS pattern of PtPdRhRuCu MMNs.

Supplementary Figure 5. HAADF-STEM image of PtPdRhRuCu MMNs showing possible atomic steps/kinks.

Supplementary Figure 6. SEM image and corresponding SEM-EDS maps of PtPdRhRuCu MMNs.

Supplementary Figure 7. Low-magnification HAADF-STEM image and corresponding map of PtPdRhRuCu MMNs.

Supplementary Figure 8. XPS spectra of PtPdRhRuCu MMNs.

Supplementary Figure 9. The time-depending SEM images for preparing PtPdRhRuCu MMNs.

Supplementary Figure 10. The optimized chemical reduction conditions.

Supplementary Figure 11. SEM images and corresponding SEM-EDS results of PtPdRhRuCu MMNs prepared at different temperature.

Supplementary Figure 12. SEM images of PtPdRhRuCu MMNs prepared using different amount of HCl.

Supplementary Figure 13. SEM images and XRD patterns of monometallic sample.

Supplementary Figure 14. SEM images and XRD patterns of mesoporous bimetallic alloy.

Supplementary Figure 15. SEM images and XRD patterns of mesoporous trimetallic alloy.

Supplementary Figure 16. SEM images andXRD patterns of mesoporous tetrametallic alloy.

Supplementary Figure 17. SEM images, N_2 adsorption–desorption isotherms, and EDS atomic maps of PtPdRhRuCu-1, PtPdRhRuCu, and PtPdRhRuCu-2 MMNs.

Supplementary Figure 18. Structural characterization of multimetallic PtPdCuNiCo alloy.

Supplementary Figure 19. Structural characterization of multimetallic PtPdCuNiCoMo alloy.

Supplementary Figure 20. Comparing HER performance of PtPdRhRuCu MMNs with other Pt-based mesoporous alloys.

Supplementary Figure 21. Powder XRD pattern of Pt MNs and PtPdRhRuCu MMNs.

Supplementary Figure 22. Electrochemical performance of PtPdRhRuCu MMNs and nonporous PtPdRhRuCu nanospheres.

Supplementary Figure 23. Electrochemical performance of PtPdRhRuCu MMNs and Pt MNs.

Supplementary Figure 24. DFT results toward HER reaction on PtPdRhRuCu MMNs.

Supplementary Figure 25. The valence electron densities of surface metal in pure metal and HEASs.

Supplementary Figure 26. The effect of pore size effect on HER performance.

Supplementary Figure 27. Structural characterization of PtPdRhRuCu-1, PtPdRhRuCu, and PtPdRhRuCu-2 samples.

Supplementary Figure 28. The characterizations of PtPdRhRuCu MMNs after HER stability test.

Supplementary Figure 29. HAADF-STEM image and corresponding map of PtPdRhRuCu MMNs after the durability HER test.

Supplementary Figure 30. Electrochemical performance in 0.5 M H_2SO_4 and1.0 M PBS electrolytes.

Supplementary Figure 31. The experimentally measured and theoretically calculated amounts of H_2 on PtPdRhRuCu MMNs in different electrolytes.

Supplementary Figure 32. Calibration curves in different electrolytes.

Supplementary Tables

Supplementary Table 1. The ICP–OES analysis of as-prepared and after alkaline HER test of PtPdRhRuCu MMNs.

Supplementary Table 2. The standard reduction potential of different half reactions for various metal precursors.

Supplementary Table 3. The feeding amounts of various metal precursors and the final compositions of Pt-based MNs.

Supplementary Table 4. The synthesis conditions for noble-mental-based MNs.

Supplementary Table 5: The calculation of Löwdin charge analysis for each metal of HEAS.

Supplementary Table 6. The HER performance of recent reported MMA electrocatalysis.

Supplementary Table 7: Summary of recently reported Pt-based HER electrocatalysts.

Supplementary References

Supplementary Figures

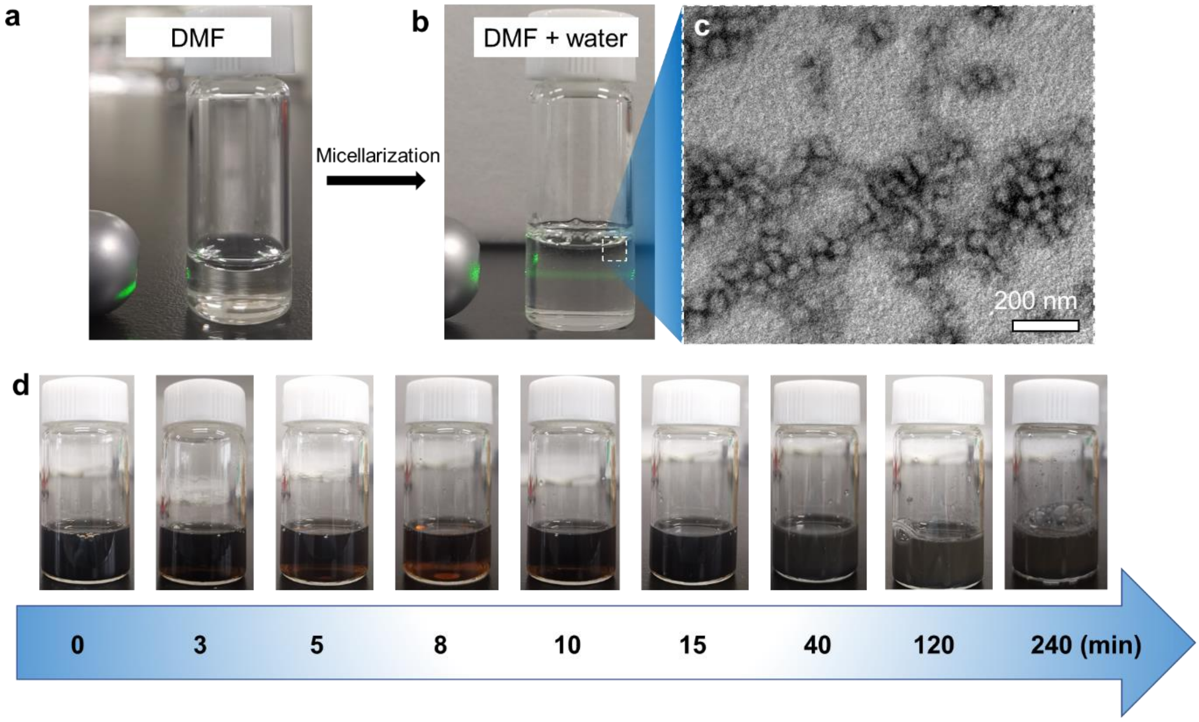

Supplementary Figure 1. (a-b) The photographs of PEO_{10500}-*b*-$PMMA_{18000}$ dissolved in DMF and following micellization process driven by adding aqueous solution, as seen through the Tyndall effect. (Note that the pure water was used instead of metal precursors because that the reaction mixture appears dark brown, leading to the optical path cannot be observed). (c) The TEM image of the spherical PEO_{10500}-*b*-$PMMA_{18000}$ micelles composed of PMMA cores surrounded by PEO shells, obtained by using a negative staining technique with phosphotungstic acid. (d) Photograph of a chemical reduction process for synthesis of PtPdRhRuCu MMNs.

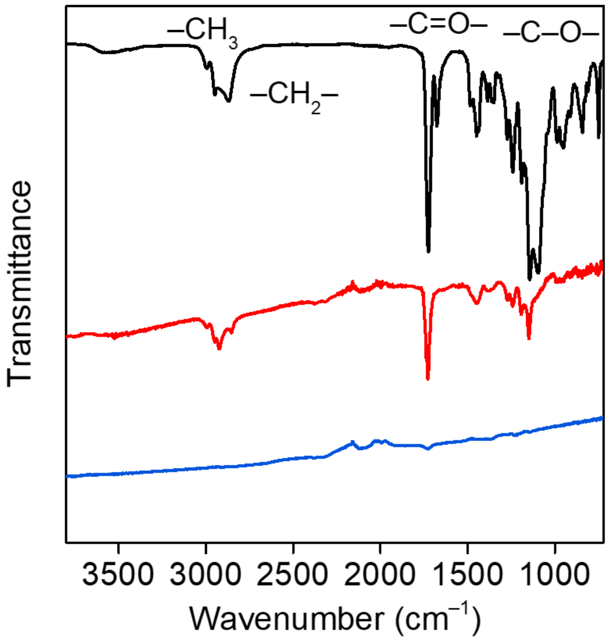

Supplementary Figure 2. Fourier transform infrared spectroscopy spectra of bare PEO_{10500}-*b*-$PMMA_{18000}$, PtPdRhRuCu MMNs before and after solvent extraction, indicating that most of the micelles were successfully removed by acetone/ethanol washing. Source data are provided as a Source Data file.

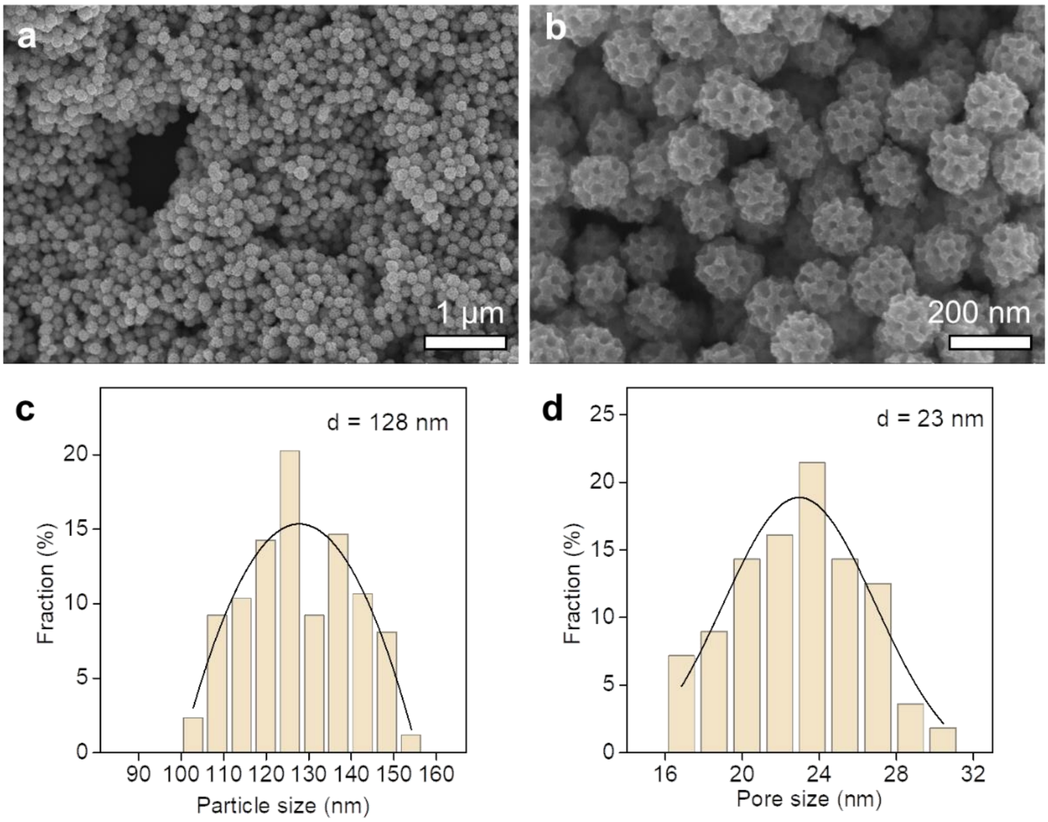

Supplementary Figure 3. (a, b) SEM images of PtPdRhRuCu MMNs. The corresponding (c) particle size and (d) pore size distribution. Source data are provided as a Source Data file.

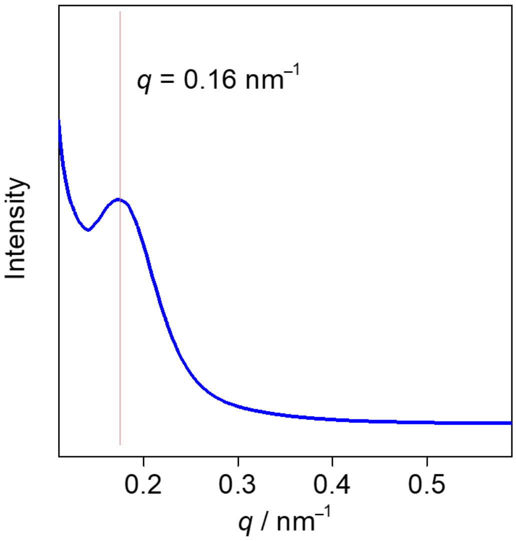

Supplementary Figure 4. The SAXS pattern of PtPdRhRuCu MMNs. (The position of each pixel on the SAXS image was converted into the scattering angle 2θ or the scattering vector q (its modulus was $q = 4\pi \sin(\theta)/\lambda$, where λ is the wavelength of the X-ray). And q was more common used because the value of θ varies in a small range.) Source data are provided as a Source Data file.

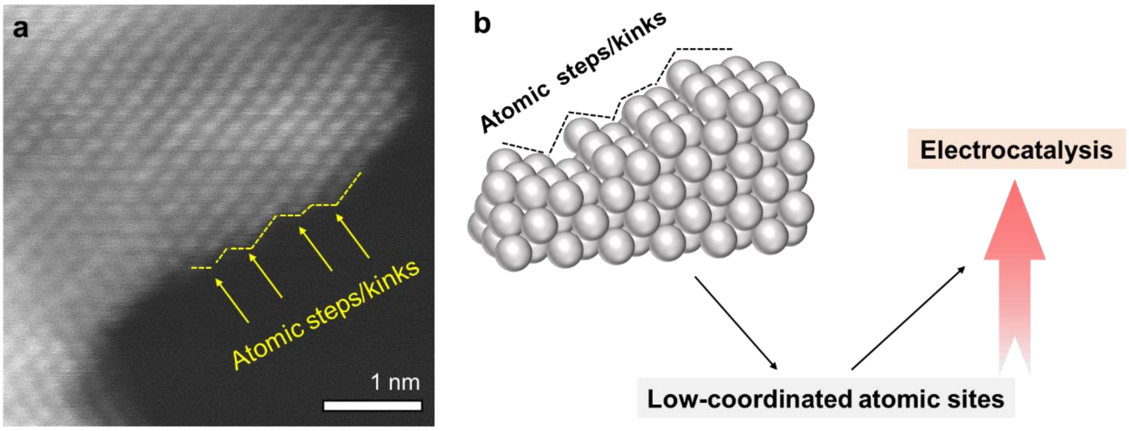

Supplementary Figure 5. (a) HAADF-STEM image of PtPdRhRuCu MMNs. (b) Schematic illustration for the atomic steps and kinks for enhancing the electrocatalytic properties.

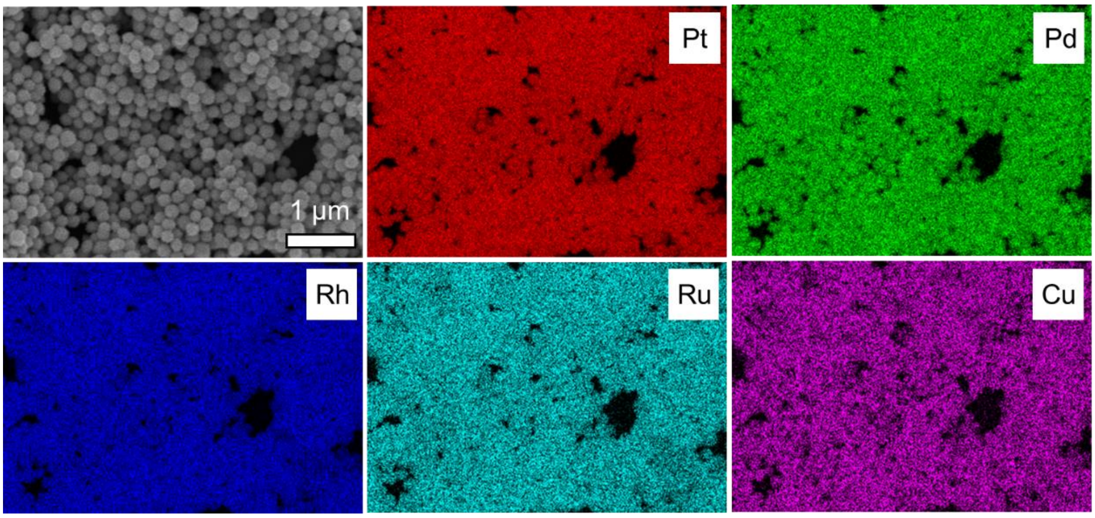

Supplementary Figure 6. SEM image and corresponding SEM-EDS maps of PtPdRhRuCu MMNs.

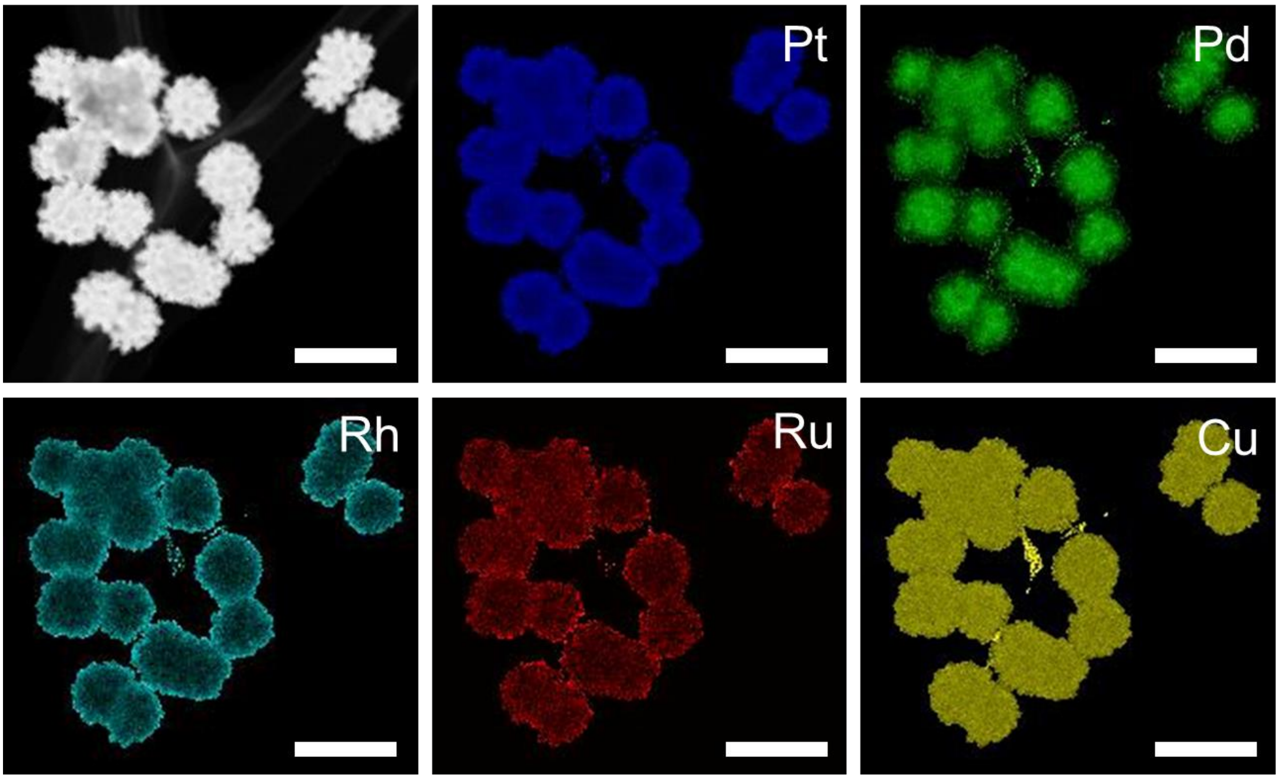

Supplementary Figure 7. Low-magnification HAADF-STEM image and corresponding map of PtPdRhRuCu MMNs. Scale bars, 200 nm.

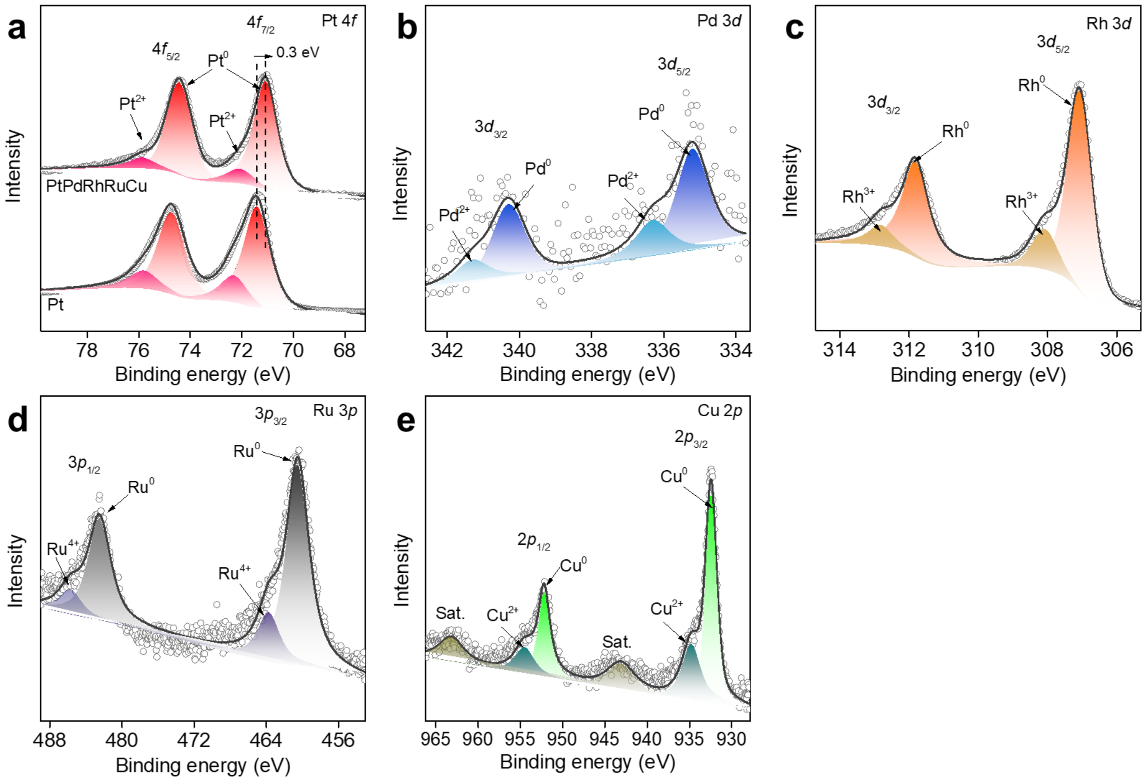

Supplementary Figure 8. (a) Pt 4*f* XPS spectra of PtPdRhRuCu and Pt MNs. (b) Pd 3*d*, Rh 3*d*, Ru 3*p* and Cu 2*p* XPS spectra of PtPdRhRuCu MMNs. Source data are provided as a Source Data file.

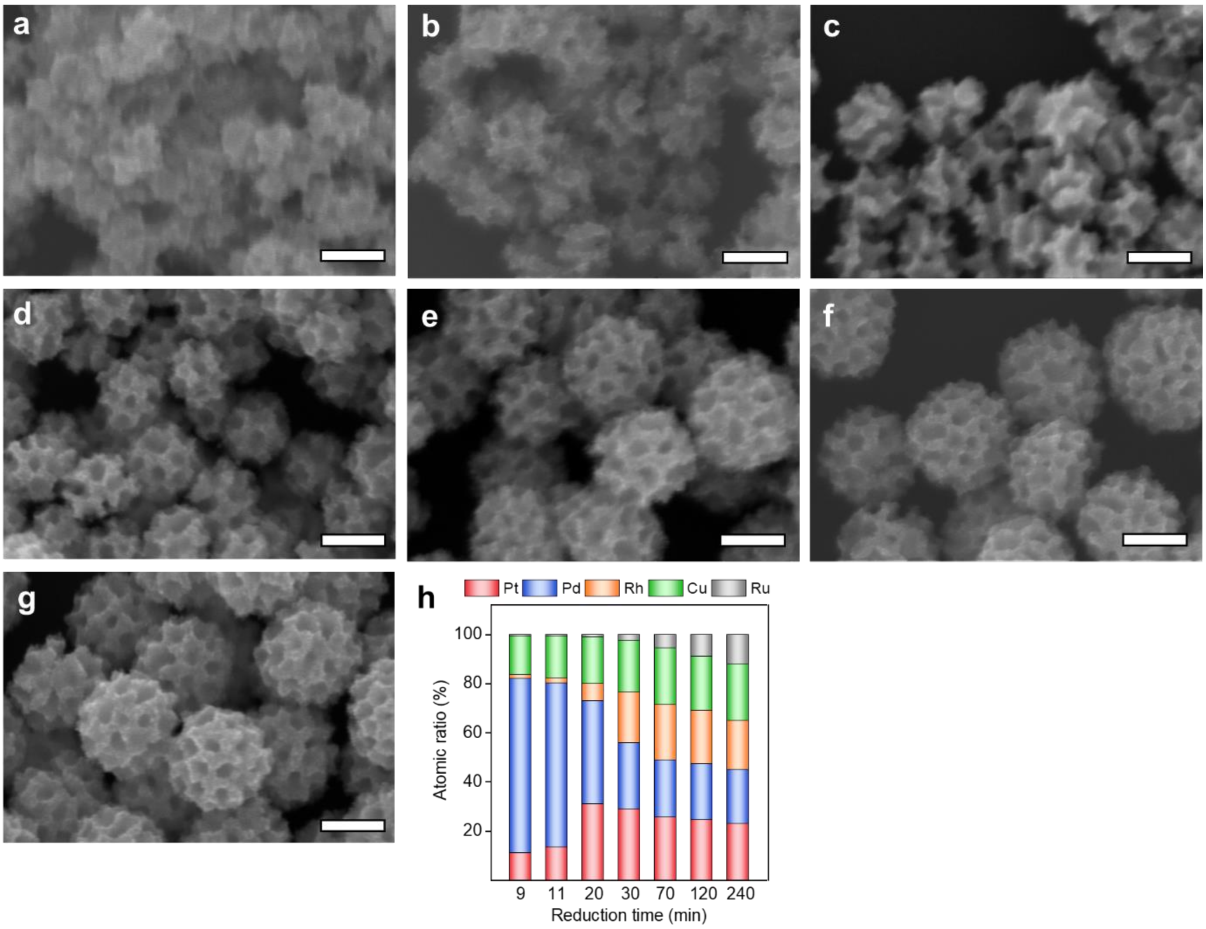

Supplementary Figure 9. The time-depending SEM images for preparing PtPdRhRuCu MMNs: (a) 9, (b) 11, (c) 20, (d) 30, (e) 70, (f) 120, and (g) 240 min. Scale bars: 100 nm (h) The changes of the composition in different deposition time, obtained by SEM-EDS. Source data are provided as a Source Data file.

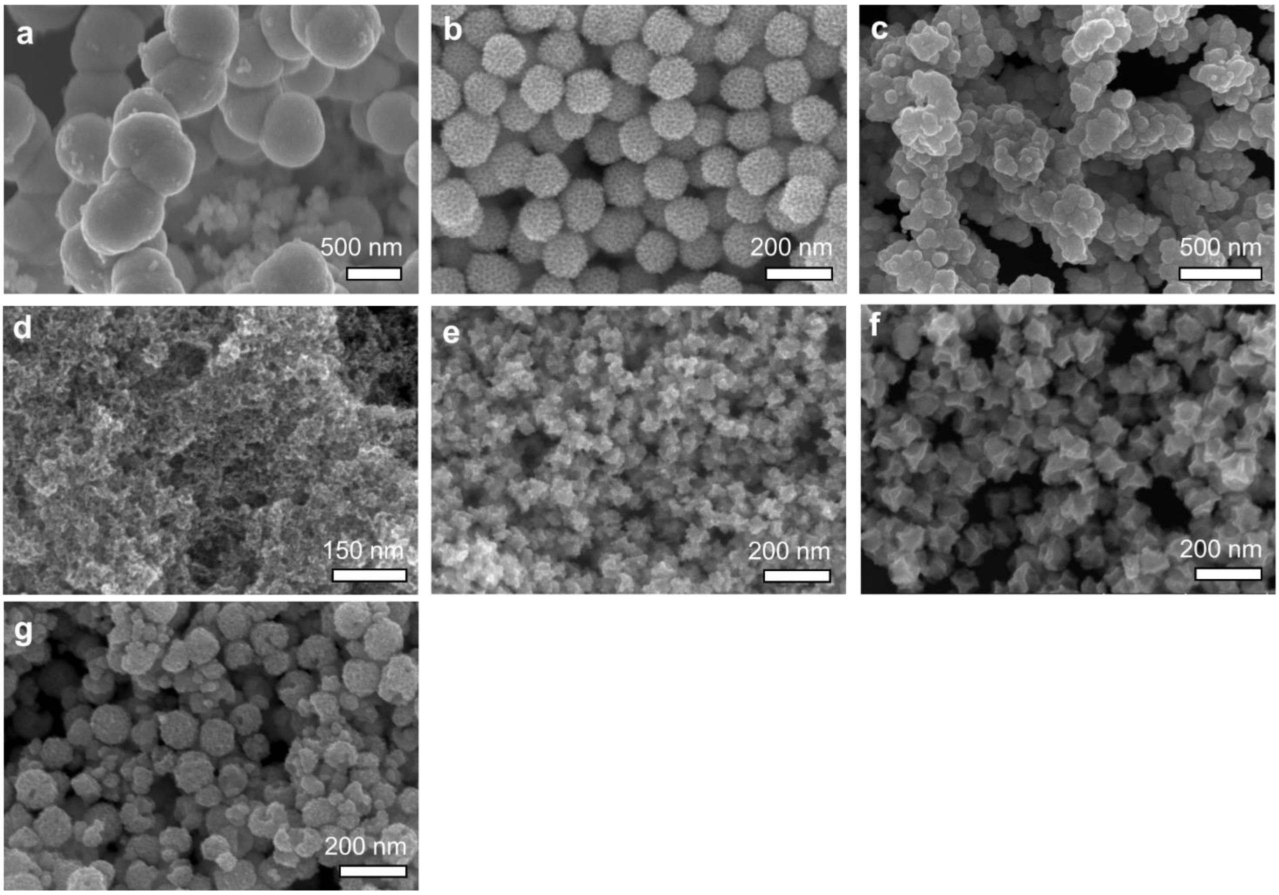

Supplementary Figure 10. The optimized chemical reduction conditions. SEM images of PtPdRhRuCu prepared (a) without using surfactant, (b) with F127, and (c) with polyvinylpyrrolidone (PVP, MW=40,000 g mol^{-1}) instead of PEO_{10500}-*b*-$PMMA_{18000}$. SEM images of PtPdRhRuCu-based samples prepared using different reducing agents: (d) dimethylamine borane and (e) formic acid. SEM images of PtPdRhRuCu samples prepared using (f) THF (3 mL) and (g) H_2SO_4 (3 M, 0.4 mL) instead of DMF (3 mL) and HCl (6 M, 0.4 mL), respectively.

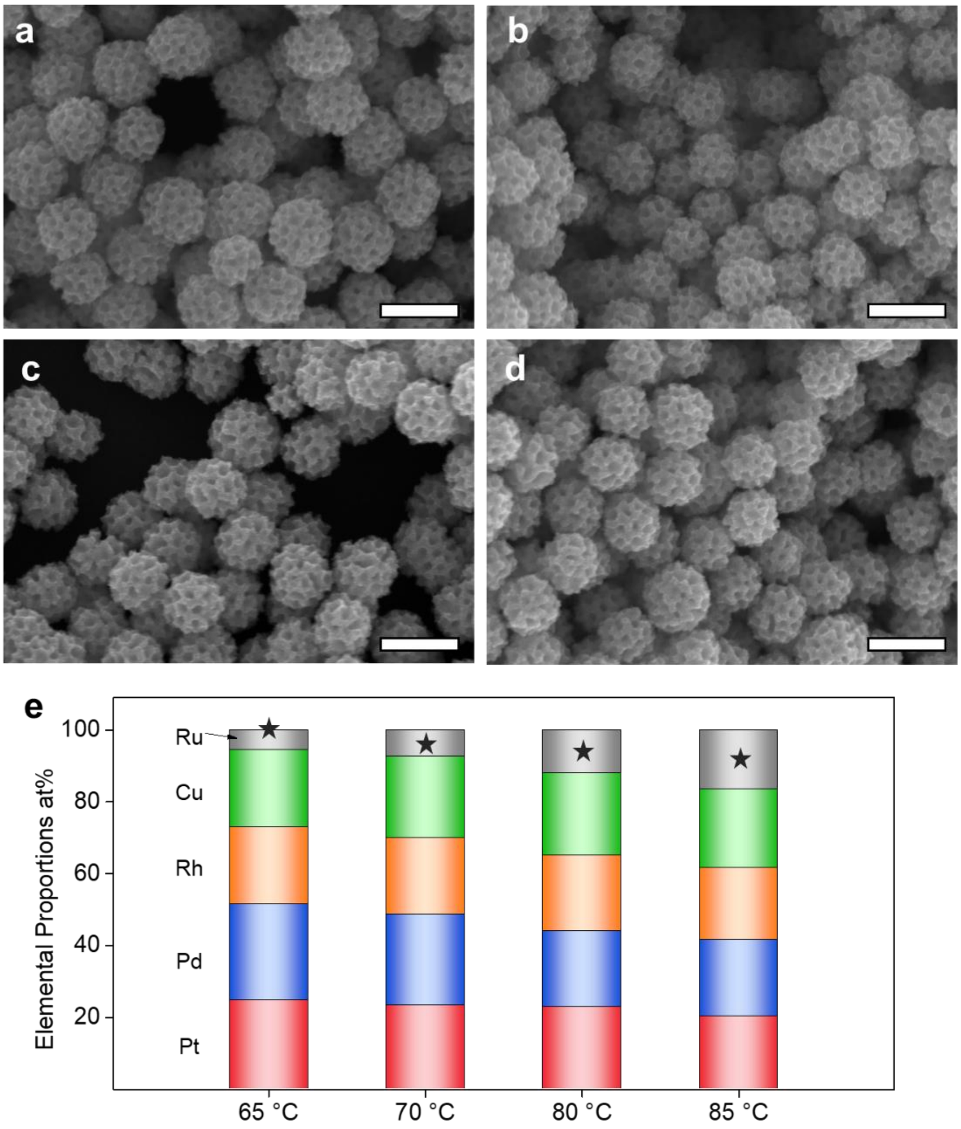

Supplementary Figure 11. (a–d) SEM images (scale bars, 200 nm.) and (e) corresponding SEM-EDS results of PtPdRhRuCu MMNs prepared at different temperature: (a) 65, (b) 75, (c) 80, and (d) 85 °C. Source data are provided as a Source Data file.

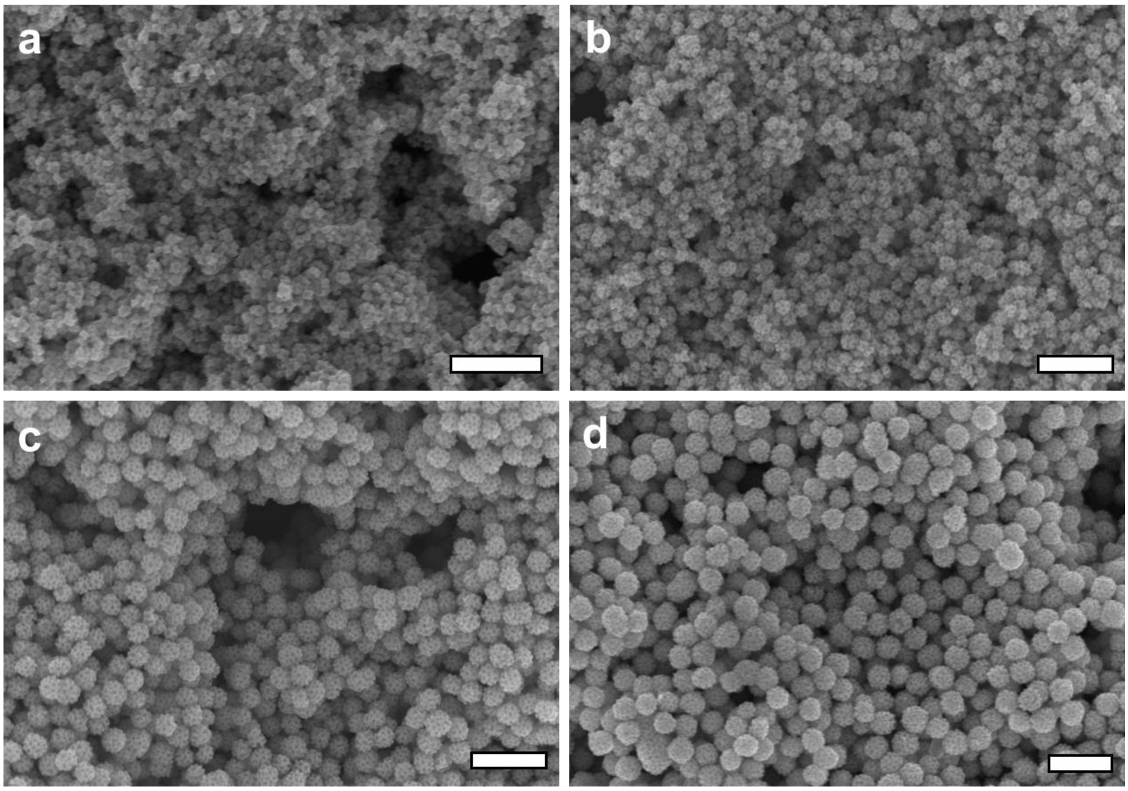

Supplementary Figure 12. SEM images of PtPdRhRuCu MMNs prepared using different amount of 6.0 M HCl: (a) 0.2, (b) 0.3, (c) 0.4, and (d) 0.5 mL. Scale bars: 500 nm.

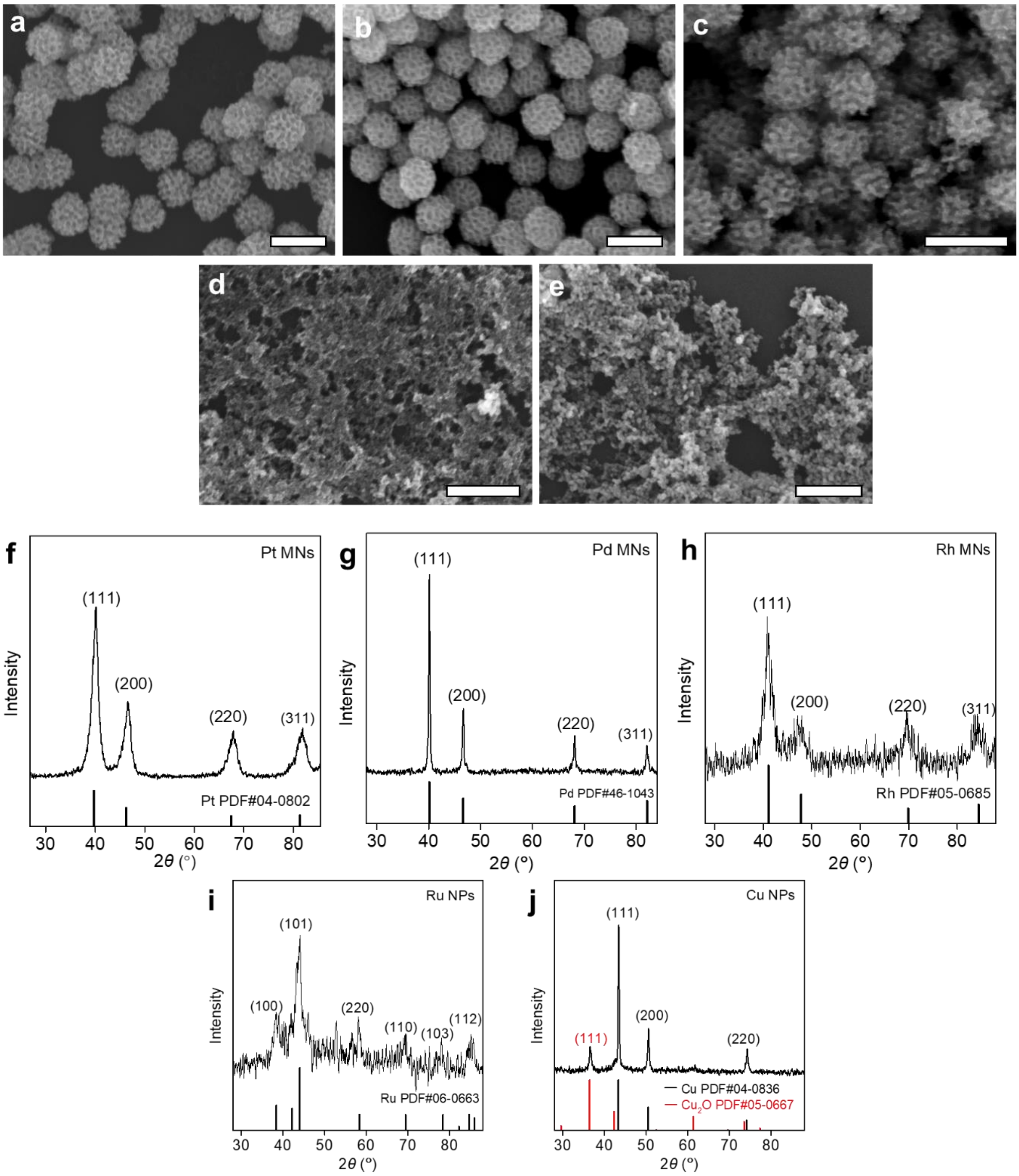

Supplementary Figure 13. SEM images and XRD patterns of monometallic sample: (a, f) Pt MNs, (b, g) Pd MNs, (c, h) Rh MNs, (d, i) Ru NPs, and (e, j) Cu NPs. Scale bars in (a–e): 200 nm. To date, mesoporous nanospheres morphology of metallic Ru and Cu were still limited to their alloys with other precious metals. The monometallic Ru and Cu with mesoporous structure were not yet successfully prepared. Therefore, Ru and Cu NPs were used instead of mesoporous structures. Source data are provided as a Source Data file.

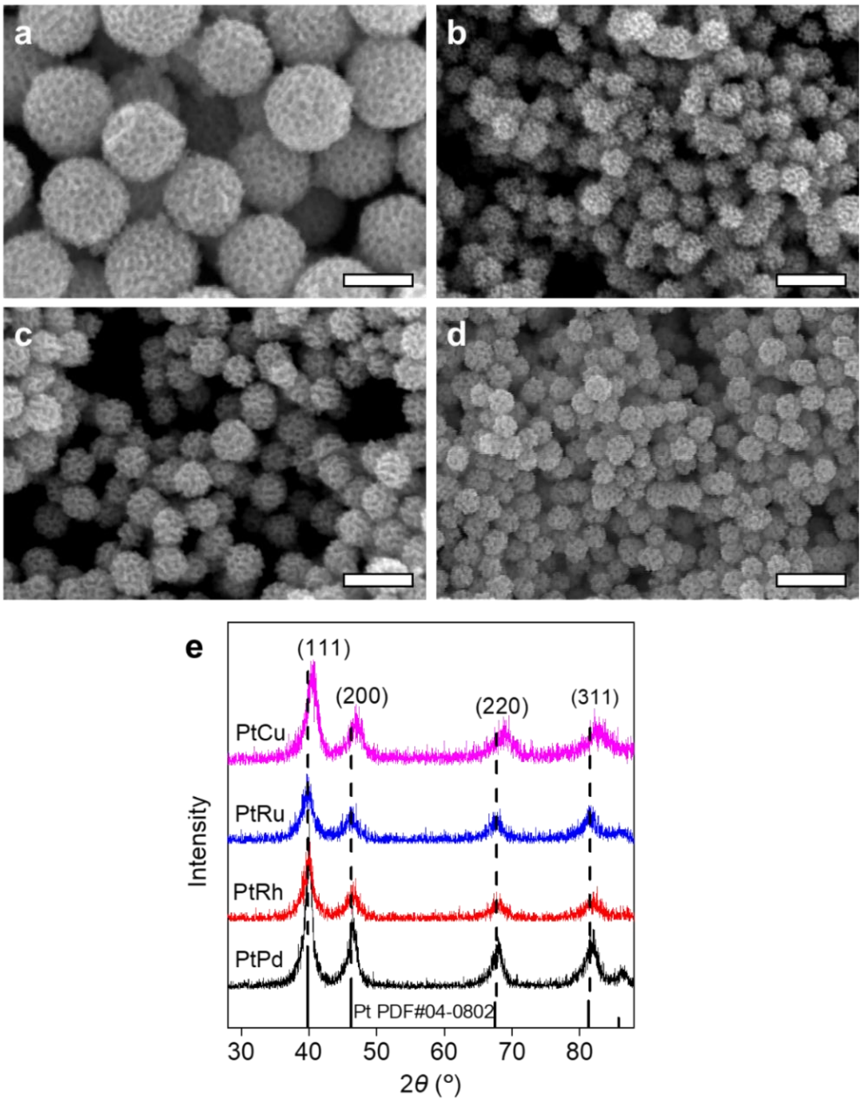

Supplementary Figure 14. SEM images of mesoporous bimetallic alloy: (a) PtPd, (b) PtRh, (c) PtCu, and (d) PtRu MNs. Scale bars: 200 nm. (e) XRD patterns of different samples. Compared to Pt (PDF#04-0802), the XRD peaks of PtPd do not significantly shift probably due to a low lattice mismatch of 0.77%.[1] However, it is interesting to note that we did not find a shift in the XRD peaks of PtRu and PtRh except for PtCu, which might be explained by the fact that Pt dominates in the alloys and the atomic radii of Ru and Rh are only slightly smaller than Pt (atomic radii order: Pt (130 pm) > Pd (128 pm) > Rh/Ru (125 pm) > Cu (117 pm).[2] Although the XRD peaks of PtRh and PtRu are not shifted, their full width at half maximum (FWHM) become larger, compared to PtPd alloy. According to Sherrer's formula, a larger FWHM represents a decrease in crystallite size[3]. The possible reason is the increase of defects caused in the introduction of Ru and Rh. Source data are provided as a Source Data file.

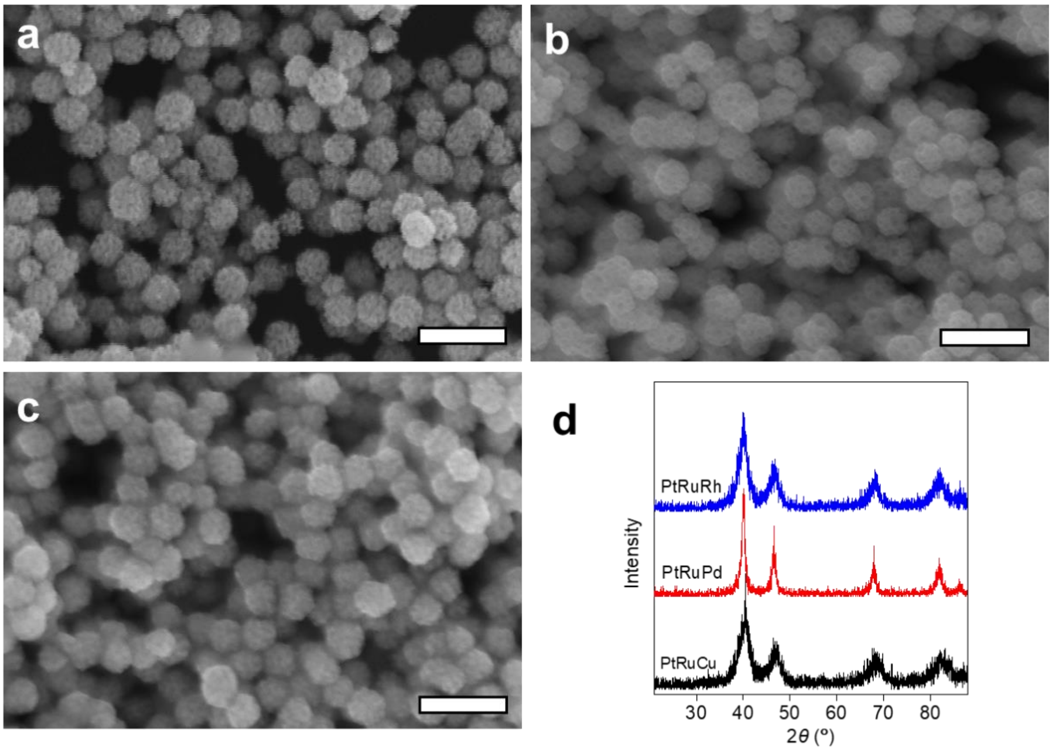

Supplementary Figure 15. (a–c) SEM images and (d) XRD patterns of mesoporous trimetallic alloy: (a) PtRuCu, (b) PtRuPd, and (c) PtRuRh MNs. Scale bars: 200 nm. Source data are provided as a Source Data file.

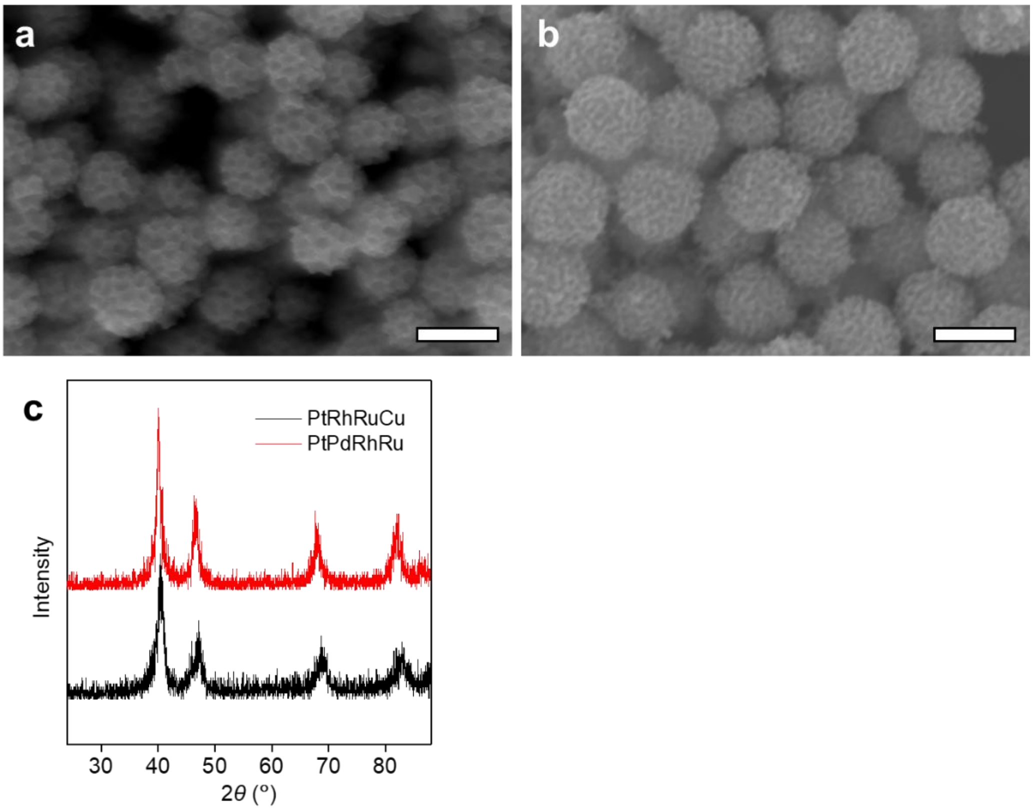

Supplementary Figure 16. (a, b) SEM images and (d) XRD patterns of mesoporous tetrametallic alloy: (a) PtRhRuCu, (b) PtPdRhRu MNs. Scale bars: 200 nm. Source data are provided as a Source Data file.

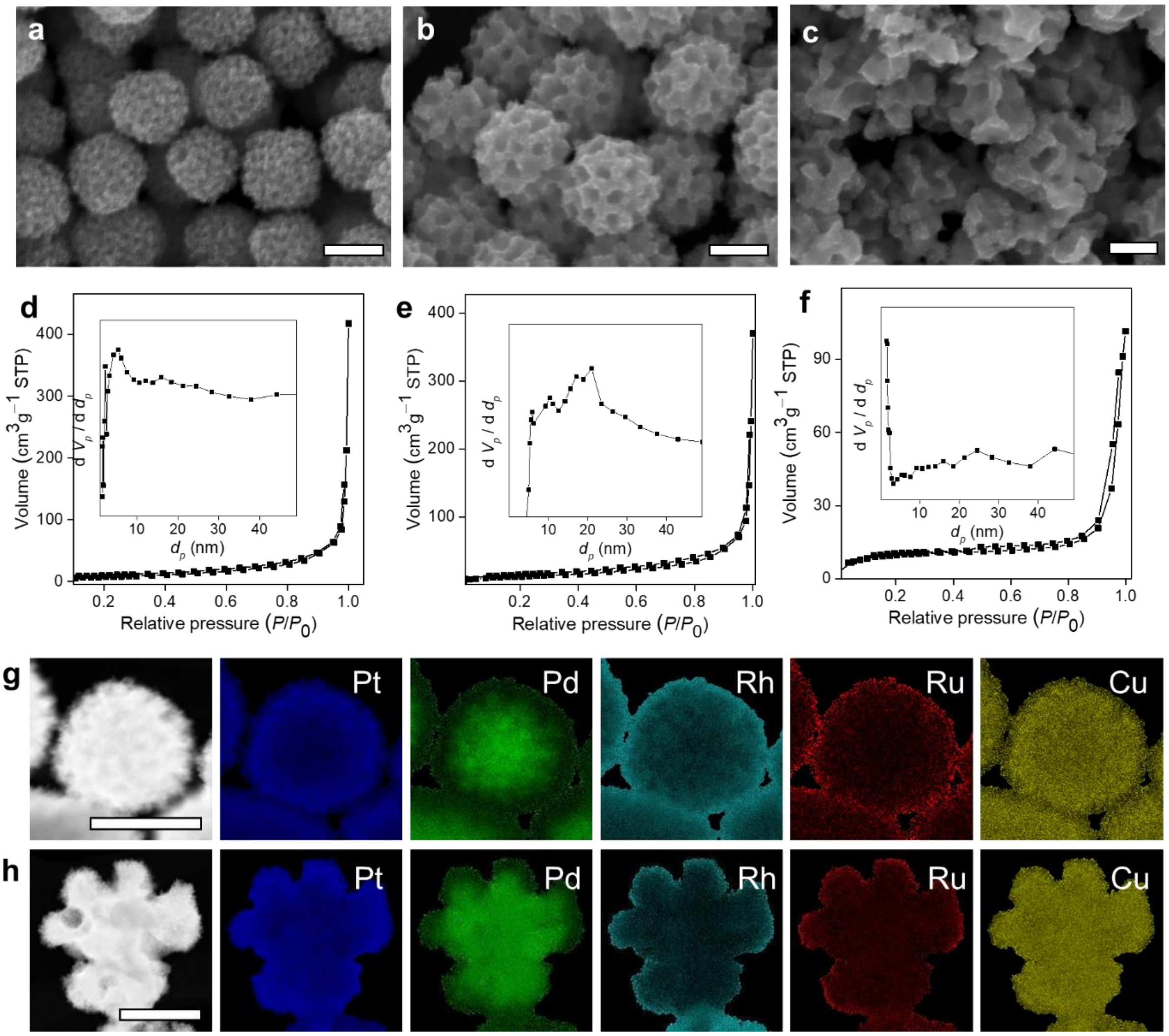

Supplementary Figure 17. SEM images and N_2 adsorption–desorption isotherms of (a, d) PtPdRhRuCu-1, (b, e) PtPdRhRuCu, and (c, f) PtPdRhRuCu-2 MMNs, which was prepared using block polymer PEO$_{10000}$-*b*-PMMA$_{5500}$, PEO$_{10500}$-*b*-PMMA$_{18000}$, and (c) PEO$_{10500}$-*b*-PMMA$_{22000}$, respectively. EDS atomic maps of (g) PtPdRhRuCu-1 and (h) PtPdRhRuCu-2 MMNs. Scale bars: 100 nm. Insets of (d-f) show the corresponding pore size distributions. Source data are provided as a Source Data file.

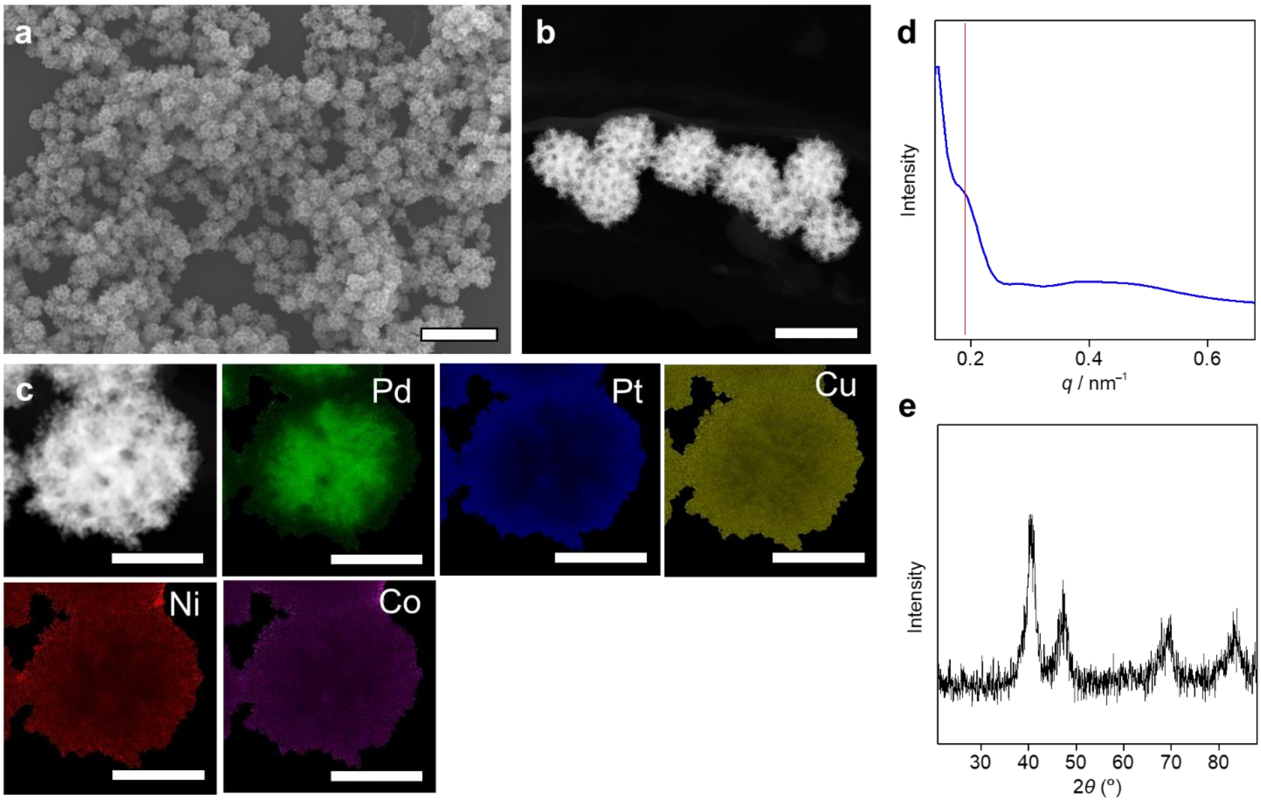

Supplementary Figure 18. (a) SEM image (Scale bar, 500 nm), (b) HAADF-STEM image (Scale bar, 100 nm), (c) EDS maps (Scale bar, 50 nm), (d) SAXS pattern, and (e) XRD pattern of multimetallic PtPdCuNiCo alloy. The atomic ratios in PtPdCuNiCo MNNs were determined to be Pt:Pd:Cu:Ni:Co = 25:28:33:7:7, based on EDS profiles. The lower molar ratio of Ni and Co probably due to their lower standard reduction potentials, which indicated that they were difficult to be reduced by *L*-AA at given experimental conditions. Source data are provided as a Source Data file.

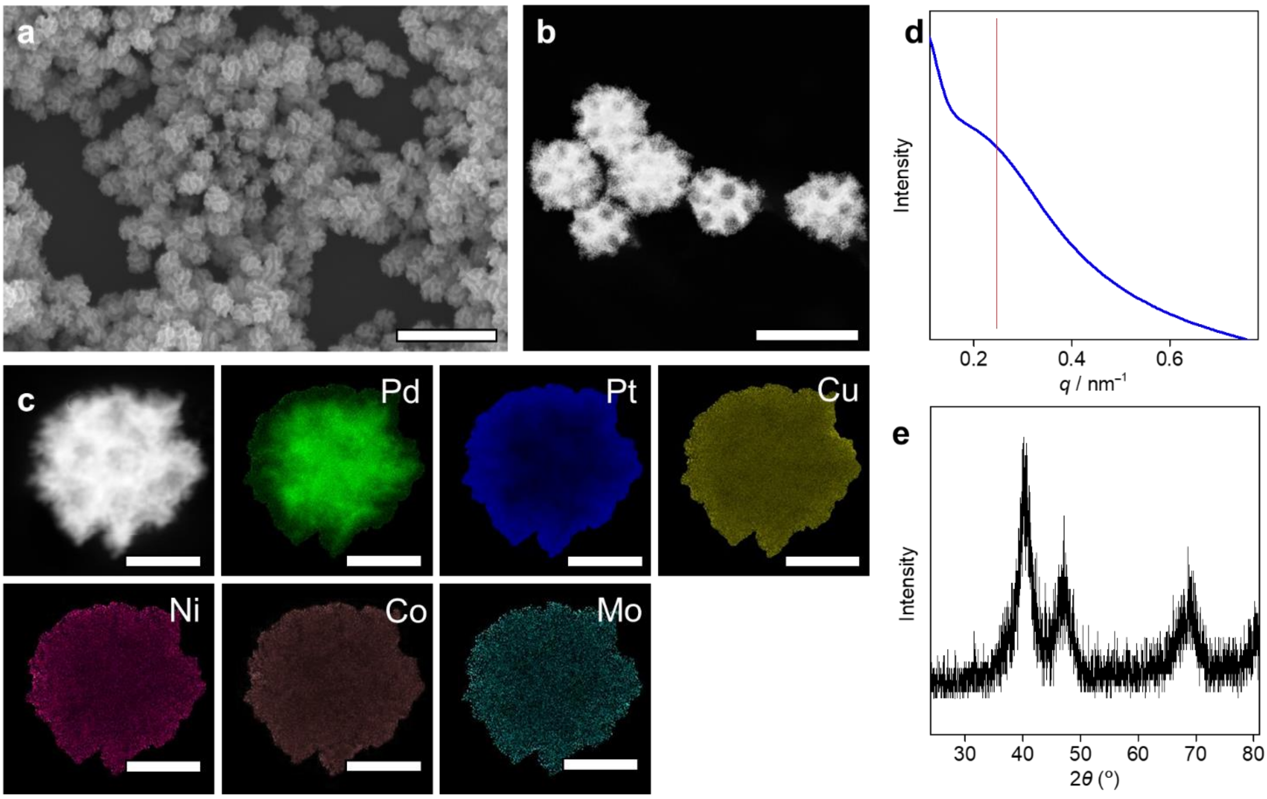

Supplementary Figure 19. (a) SEM image (Scale bar, 500 nm), (b) HAADF-STEM image (Scale bar, 100 nm), (c) EDS maps (Scale bar, 50 nm), (d) SAXS pattern, and (e) XRD pattern of multimetallic PtPdCuNiCoMo alloy. The atomic ratios in PtPdCuNiCoMo MNNs were determined to be Pt:Pd:Cu:Ni:Co:Mo = 23:24:28:10:9:6, based on EDS profiles. The lower molar ratio of Ni, Co, and Mo probably due to their lower standard reduction potentials, which indicated that they were difficult to be reduced by *L*-AA at given experimental conditions. Source data are provided as a Source Data file.

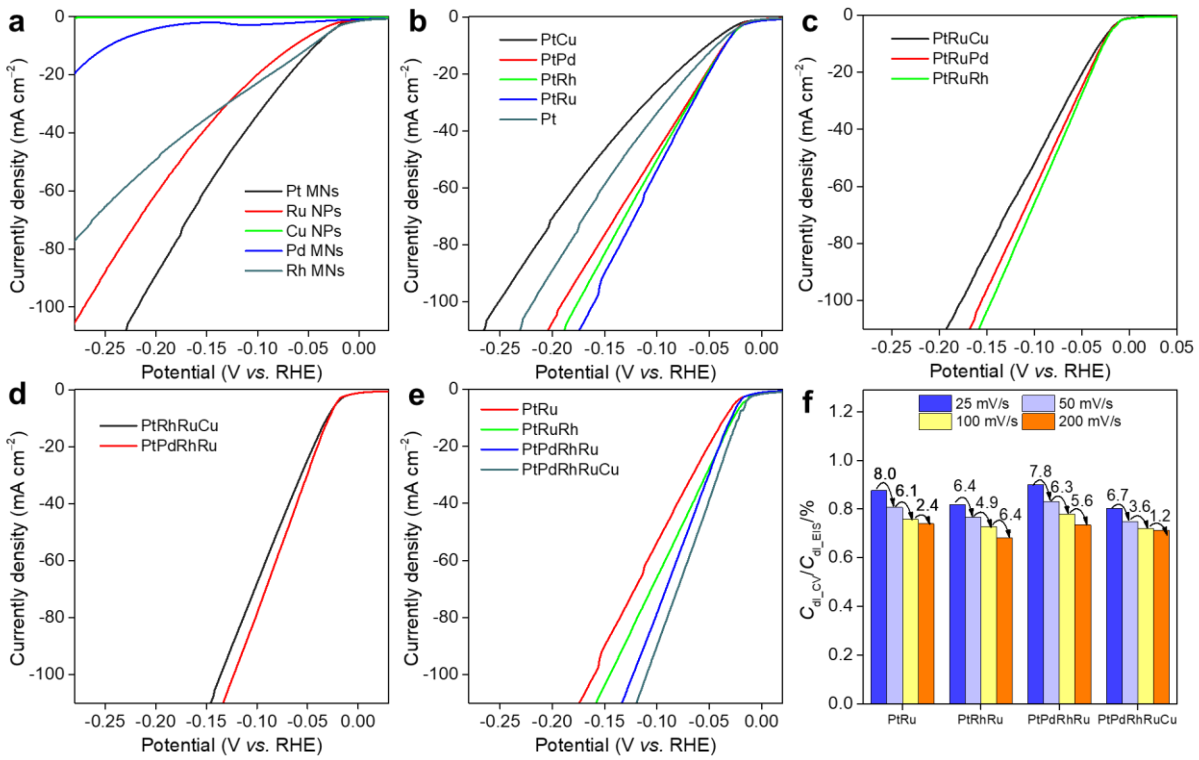

Supplementary Figure 20. HER polarization curves of (a) monometallic MNs/NPs, (b) bimetallic, (c) trimetallic, (d) tetrametallic, and (e) comparing PtPdRhRuCu MMNs with other Pt-based mesoporous alloys at scan rate of 5 mV s^{-1} without *iR* correction in 1.0 M KOH electrolyte. (e) The decline ratio between C_{dl_CV} and C_{dl_EIS} at scan rates of 25, 50, 100, and 200 mV s^{-1} of different samples. Source data are provided as a Source Data file.

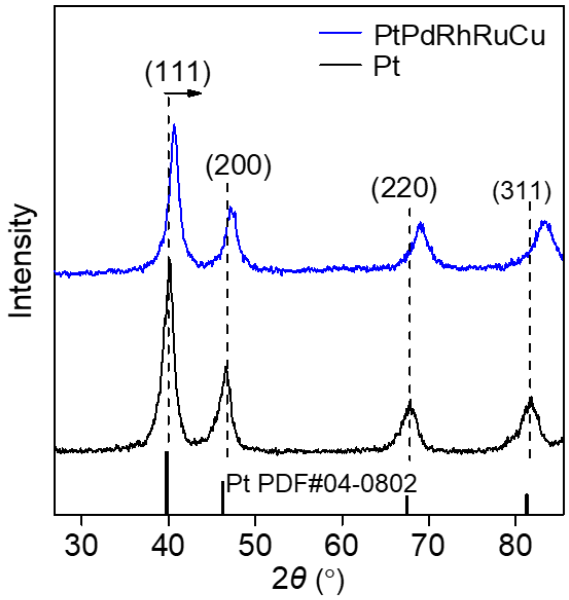

Supplementary Figure 21. Powder XRD pattern of Pt MNs and PtPdRhRuCu MMNs. Source data are provided as a Source Data file.

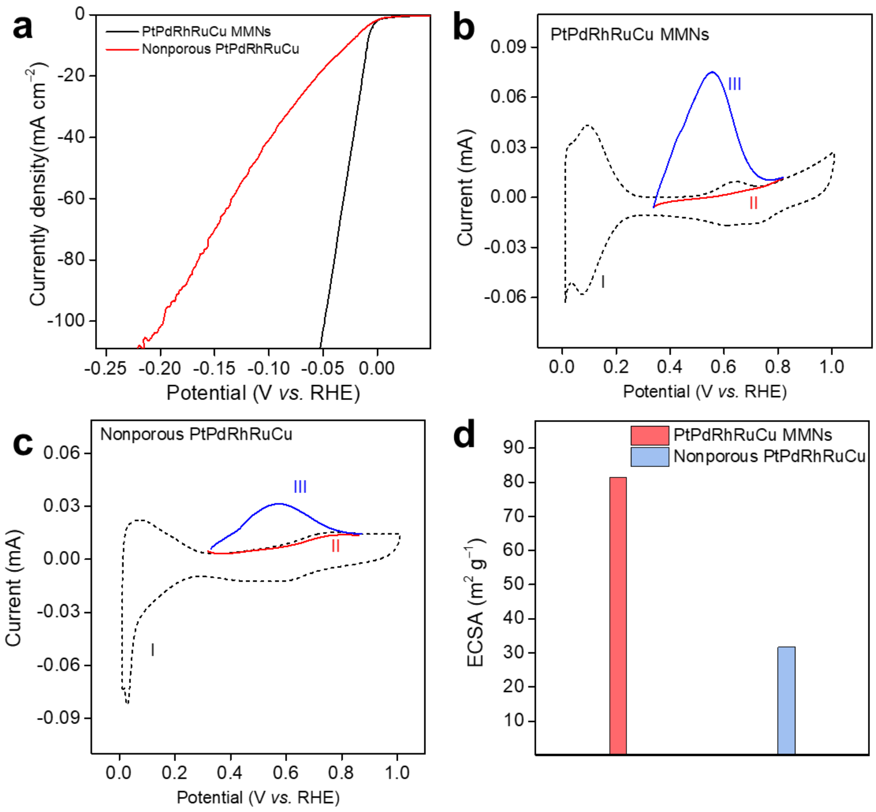

Supplementary Figure 22. (a) Polarization curves of PtPdRhRuCu MMNs and nonporous PtPdRhRuCu nanospheres. Cu-UPD measurement in 0.5 M H_2SO_4 with (I, II) and without (III) of 5 mM $CuSO_4$ on (b) PtPdRhRuCu MMNs and (c) nonporous PtPdRhRuCu nanospheres at a scan rate of 10 mV/s. The electrodes for II and III were fixed at certain deposition potential for 100 s to form UPD layer and followed by a CV scan. (f) Histograms showing the ECSA values. Source data are provided as a Source Data file.

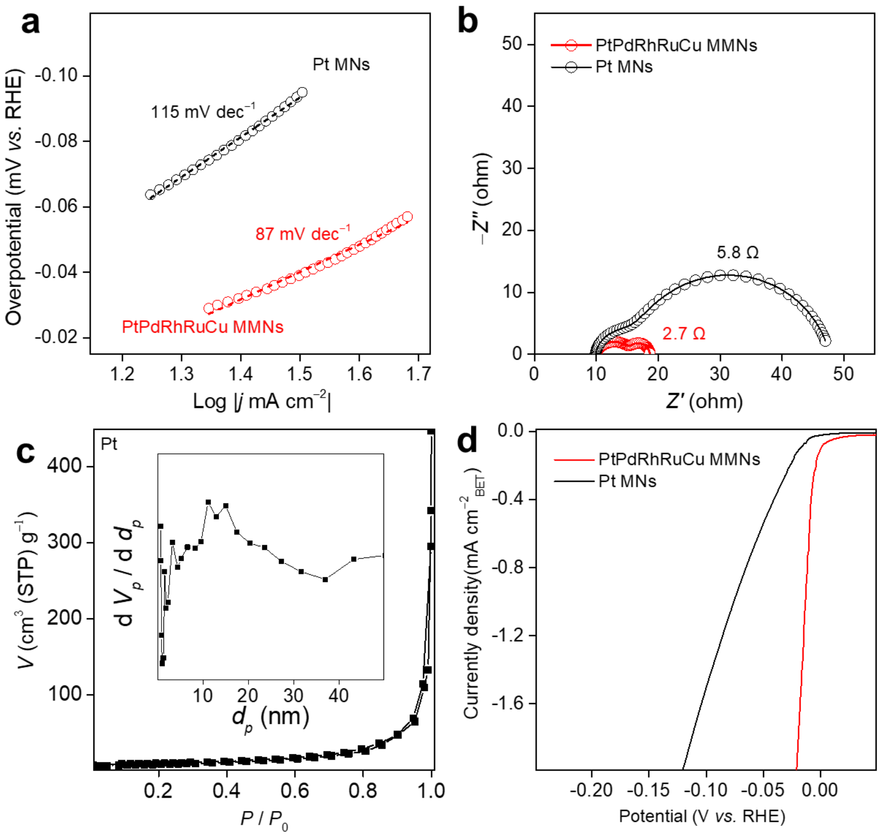

Supplementary Figure 23. (a) Tafel plots, (b) Nyquist plots (operated at the overpotential of 20 mV) of PtPdRhRuCu MMNs and Pt MNs. (c) N_2 adsorption–desorption isotherms of Pt MNs (Inset shows the corresponding pore size distribution). (d) BET-normalized LSV curves of PtPdRhRuCu MMNs and Pt MNs. Source data are provided as a Source Data file.

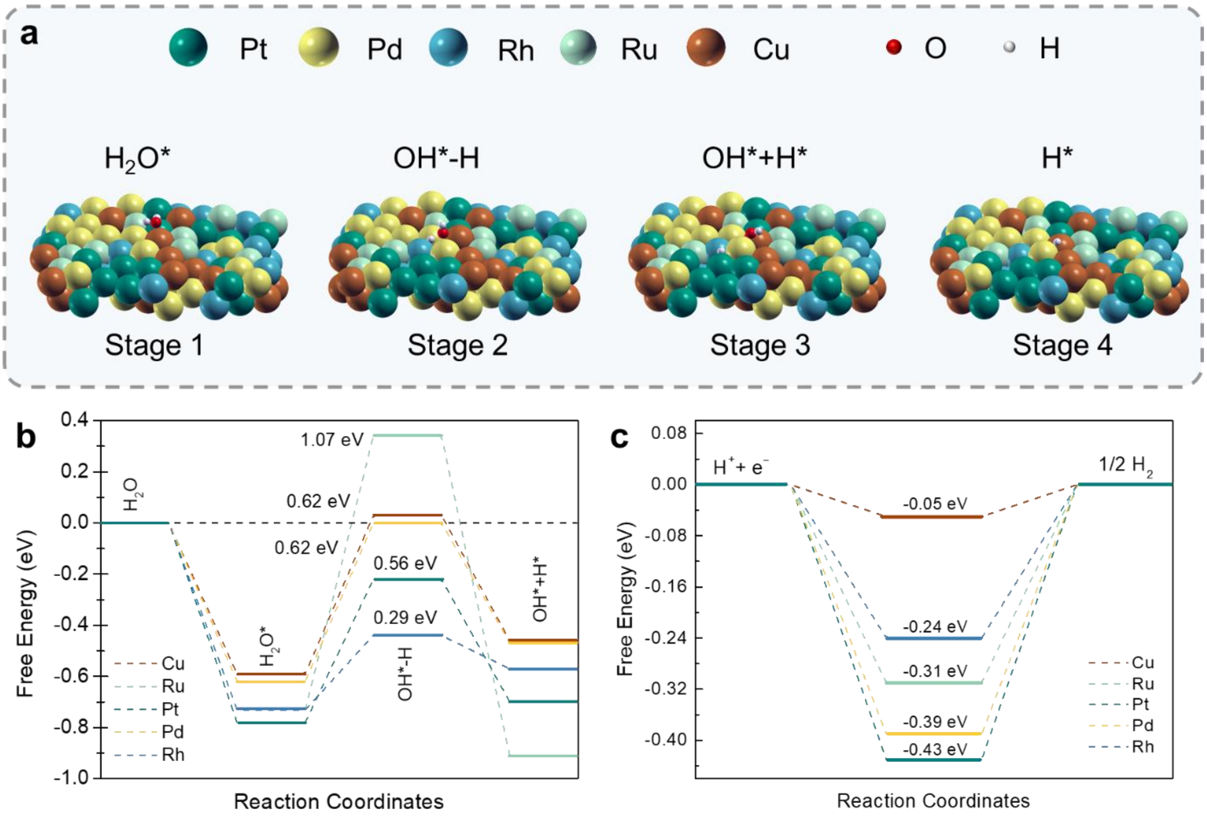

Supplementary Figure 24. (a) The atomic configurations at the four stages toward HER reaction on PtPdRhRuCu MMNs. The calculated free energy (ΔG) for (b) water dissociation and (c) hydrogen adsorption at various catalytic sites of PtPdRhRuCu. DFT calculations were performed to understand the superior HER performance of PtPdRhRuCu MMNs in alkaline media. Based on the experimentally measured Tafel slope value (87 mV dec^{-1}), the HER reaction mechanism of PtPdRhRuCu MMNs in alkaline solution might follow the Volmer-Heyrovsky mechanism (*eqs* (i-ii)).

Volmer step: $H_2O + e^- + * \rightarrow H* + OH^-$ (i)

Heyrovsky step: $H* + H_2O + e^- \rightarrow H_2 + OH^-$ (ii)

where * represented the catalytic sites on the surface; H* was the adsorbed hydrogen intermediate.

Unlike HER in acidic media, water dissociation (Volmer step) was a key step to provide the free proton for the HER reaction in alkaline solution. Pt presented a superior HER performance in acidic environment but had a much lower reaction rate in alkaline solution.[4] An ideal alkaline HER electrocatalyst should combine an optimal free energy of hydrogen adsorption (ΔG_{H*}) and a low water dissociation barrier. **Supplementary Figure 24a** displays the atomic configurations of the four elemental stages during alkaline HER at the catalytic sites of $Pt_{23}Pd_{22}Rh_{20}Ru_{13}Cu_{22}$ (The atomic ratios

of each metal was based on ICP−OES, which was close to the HEASs in PtPdRhRuCu MMNs (**Figure 2**)): (Stage 1) the adsorption of H_2O^* molecule; (Stage 2) the destabilization of adsorbed H_2O^* molecule to form H−OH bond; (Stage 3) the dissociated H−OH to generate H* and OH* intermediates; (Stage 4) the adsorbed H* will react with proton to produce H_2. As shown in the energy profile of **Supplementary Figure 24b**, in the energy barrier to water dissociation on diverse active sites of $Pt_{23}Pd_{22}Rh_{20}Ru_{13}Cu_{22}$ Rh site has the lowest barrier of 0.29 eV to break the H−OH bond (*i.e.*, stage 1 →stage 2). As a key step of HER reaction in basic environment, the energy cost of 0.29 eV for water dissociation was much lower than the 0.89 eV occurred on the pure Pt (111) surface,[5] indicating a facilitated water dissociation on the synthesized MMA of $Pt_{23}Pd_{22}Rh_{20}Ru_{13}Cu_{22}$.

The evolution of hydrogen intermediate on the catalyst for HER was usually descripted by hydrogen adsorption free energies ΔG_{H^*}, and the optimal value of ΔG_{H^*} should be close to zero (*i.e.*, $|\Delta G_{H^*}| = 0$).[6] In HER, Pt (111) was reported as an efficient HER catalyst due to its near-zero value of $|\Delta G_{H^*}| = 0.09$ eV.[5] From the calculated ΔG_{H^*} on different sites of *fcc* $Pt_{23}Pd_{22}Rh_{20}Ru_{13}Cu_{22}$ (**Supplementary Figure 24c**), Cu site has a much lower $|\Delta G_{H}*|$ value of 0.05 eV than the reported result on Pt (111). The synergistic effect between the active metallic site of MMA, which has altered the surface reactivity to hydrogen adsorption, was common in metal alloy or hybrid catalysts.[7, 8, 9, 10] For example, as reported by Li and coworkers,[8] the nearby Ni, Co sites in $Pt_{18}Ni_{26}Fe_{15}Co_{14}Cu_{27}$ catalyst were considered as the preferred sites for H* adsorption to stabilize H in the alkaline HER. The localized effect of each metal coupled to its neighboring metals in MMA catalysts lead to a more chemical complexity on the catalytic surface of MMA.[11, 12] Therefore, the catalytic performance of PtPdRhRuCu MMNs with multi-metal involved is not simply dependent on the each separate metal, but on a cooperative effect from the active metal site and its coordinated metals, which is beneficial to a pH-universal HER catalytic activity and stability. Source data are provided as a Source Data file.

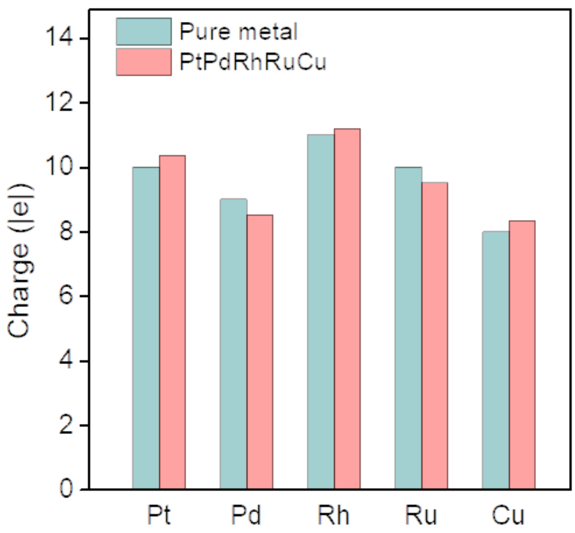

Supplementary Figure 25. The valence electron densities of surface metal in pure metal and HEASs of PtPdRhRuCu MMNs respectively from Löwdin charge analysis, which was study the charge transfer effects. The change of electronic structure might change Fermi level and the *d*-band center position of metals, thus optimized the adsorption for the reaction intermediates to influence the electrocatalytic activity.[13] Source data are provided as a Source Data file.

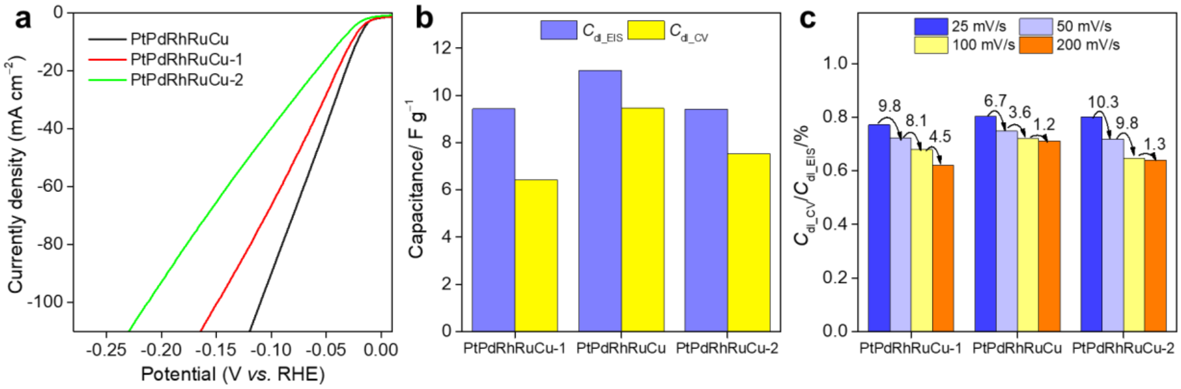

Supplementary Figure 26. (a) Polarization curves of HER without *iR* correction of PtPdRhRuCu-1, PtPdRhRuCu, and PtPdRhRuCu-2 MMNs. (b) The ECSA/EWSA ratio (dynamic (CV)/static (EIS) capacitance and (c) the ratio between C_{dl_CV} and C_{dl_EIS} at scan rates of 25, 50, 100, and 200 mV s^{-1} of different samples (The numbers at the top of the histogram in (c) indicate decrease rate (%) of different scan rate). To investigate the effect of the role of each kind of mesopore on performance, we conducted in-depth electrochemical analysis with EIS. The electric double layer capacitance (C_{dl}) was calculated from EIS and cyclic voltammetry (CV) to obtain C_{dl_EIS} and C_{dl_CV}, which were proportional to EWSA and ECSA, respectively. The high C_{dl_CV}/C_{dl_EIS} values and low decrease rate at increased scan rates indicate the efficient ion migration. Source data are provided as a Source Data file.

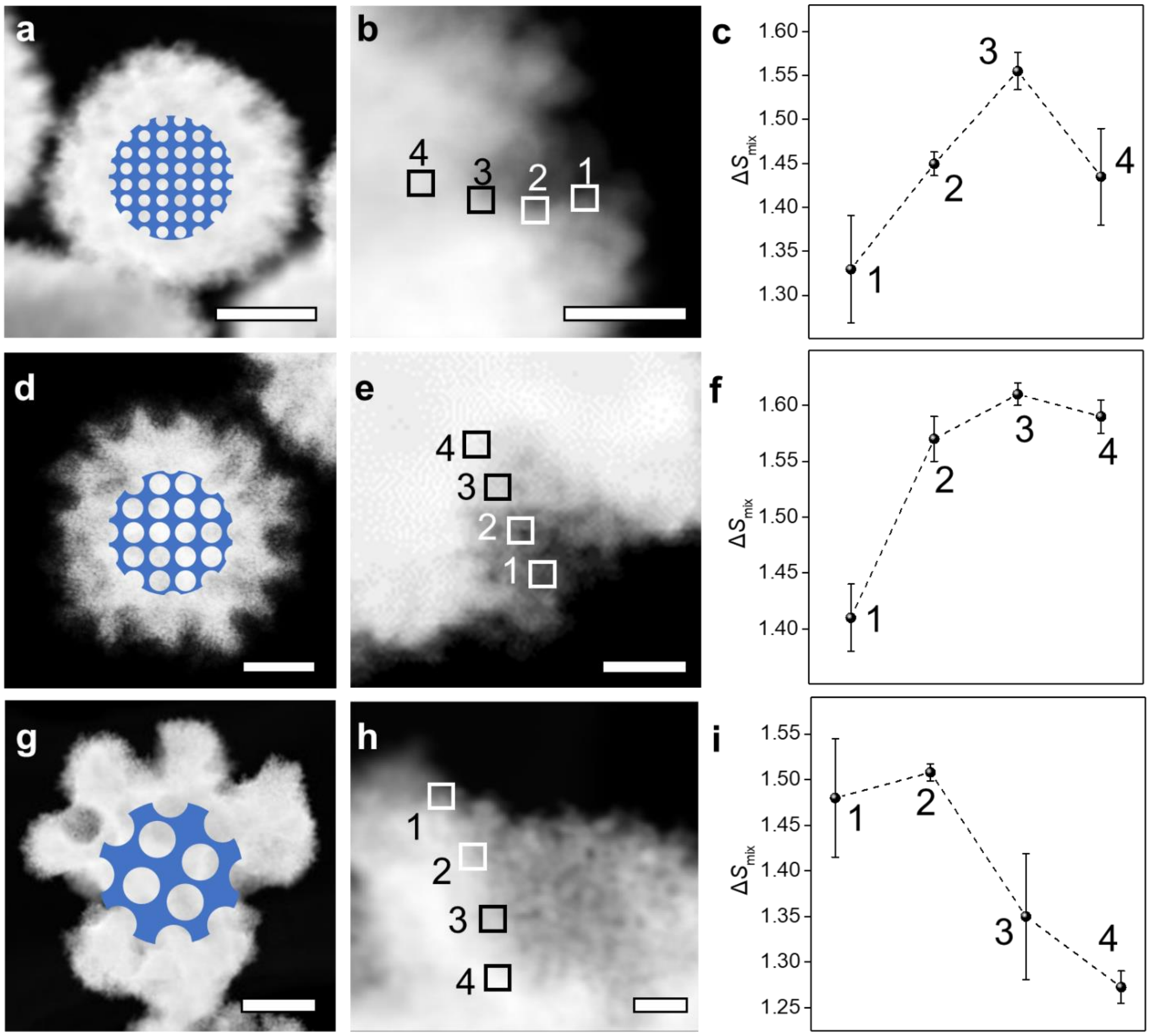

Supplementary Figure 27. (a, d, g) HAADF-STEM images, (b, e, h) enlarged image of one pore area, and (c, f, i) the corresponding ΔS_{mix} value at the selected area (i.e., 1, 2, 3, and 4) of (a–c) PtPdRhRuCu-1, (d–f) PtPdRhRuCu, and (g–i) PtPdRhRuCu-2 samples. Corresponding cartoon structure drawings are shown inset of (a, d, g). Error bars in (c, f, i) based on the measurements at three points within the selected region. Scale bars: (a, d, g) 50 nm; (b, e, h) 10 nm. Source data are provided as a Source Data file.

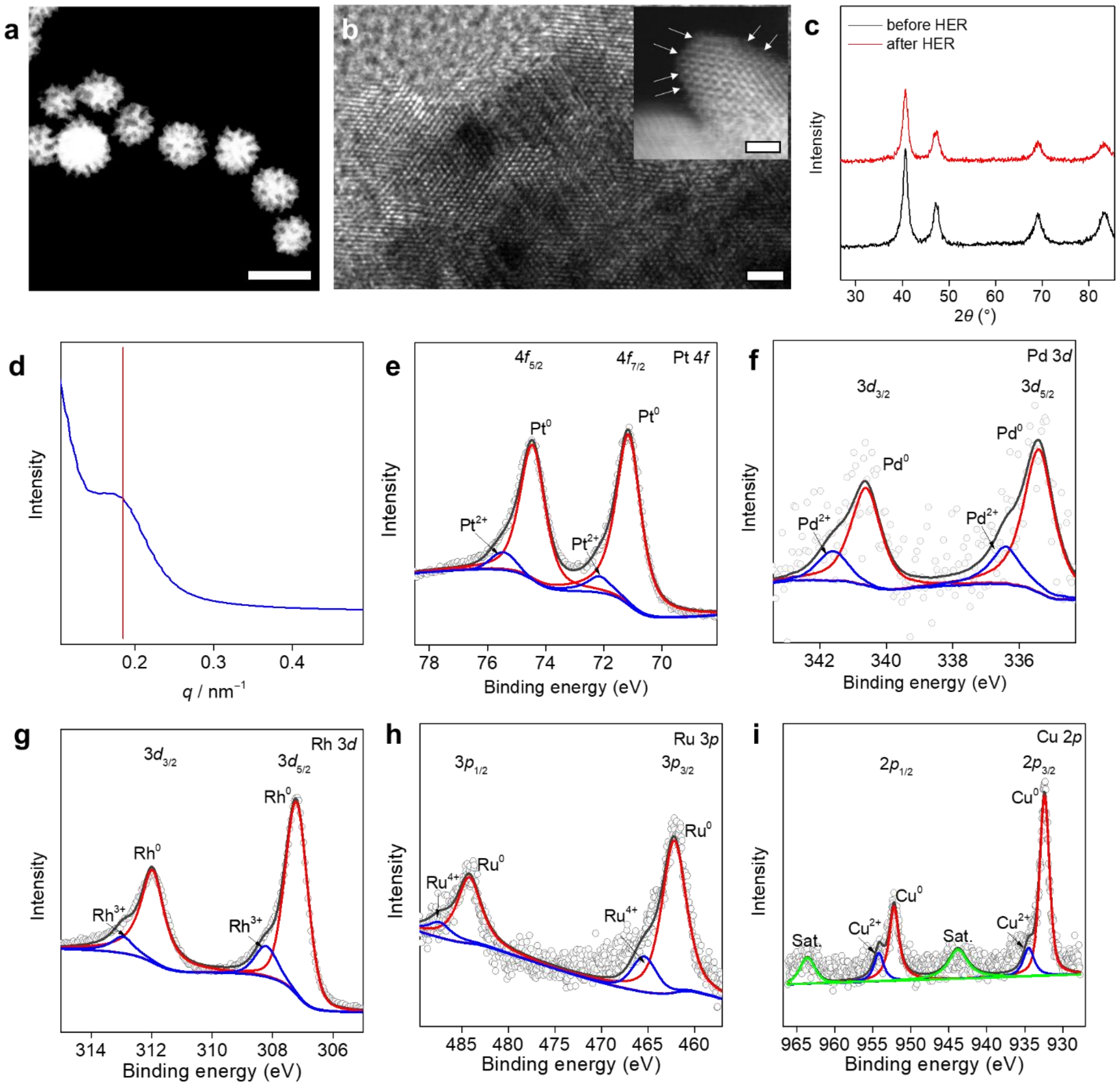

Supplementary Figure 28. The characterizations of (a) HAADF-STEM (scale bar, 200 nm), (b) HRTEM (scale bar, 2 nm), (c) XRD, (d) SAXS pattern, and (e–i) XPS spectra of PtPdRhRuCu MMNs after the durability HER test in 1.0 M KOH. Inset of (b) shows the HAADF-STEM image and as marked possible atomic steps/kinks (scale bar, 1 nm). The mesoporous morphology of PtPdRhRuCu MMNs were maintained after HER test. The atomic steps and kinks were still observed in **Supplementary Figure 28b**, which might be a contributing factor for the stability. However, considering that local surface changes on the atomic scale on nanomaterials probably occurred during the electrochemical testing, rearrangement of surfaces of the electrocatalysts was not uncommon. The stable bulk morphology, composition, and porous structure of the nanosphere might be the main factor for maintaining long-term HER measurement. Source data are provided as a Source Data file.

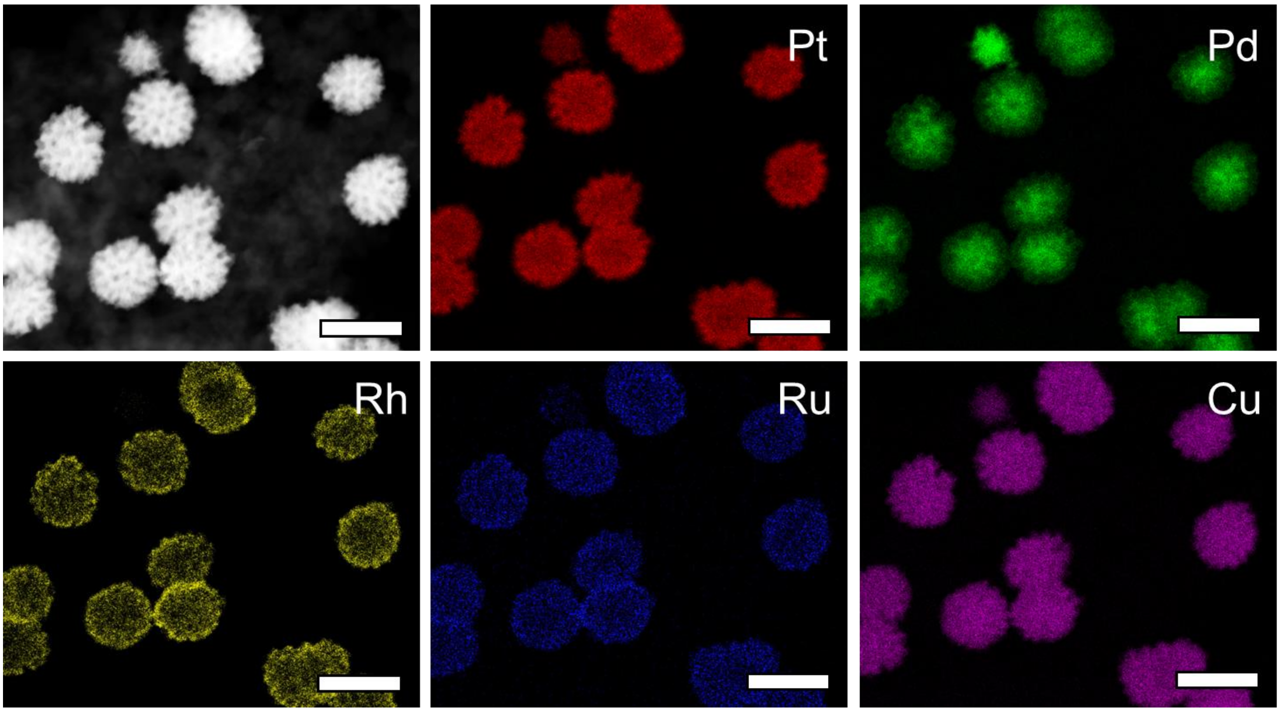

Supplementary Figure 29. HAADF-STEM image and corresponding map of PtPdRhRuCu MMNs mixed with carbon after the durability HER test in 1.0 M KOH. Scale bars, 200 nm.

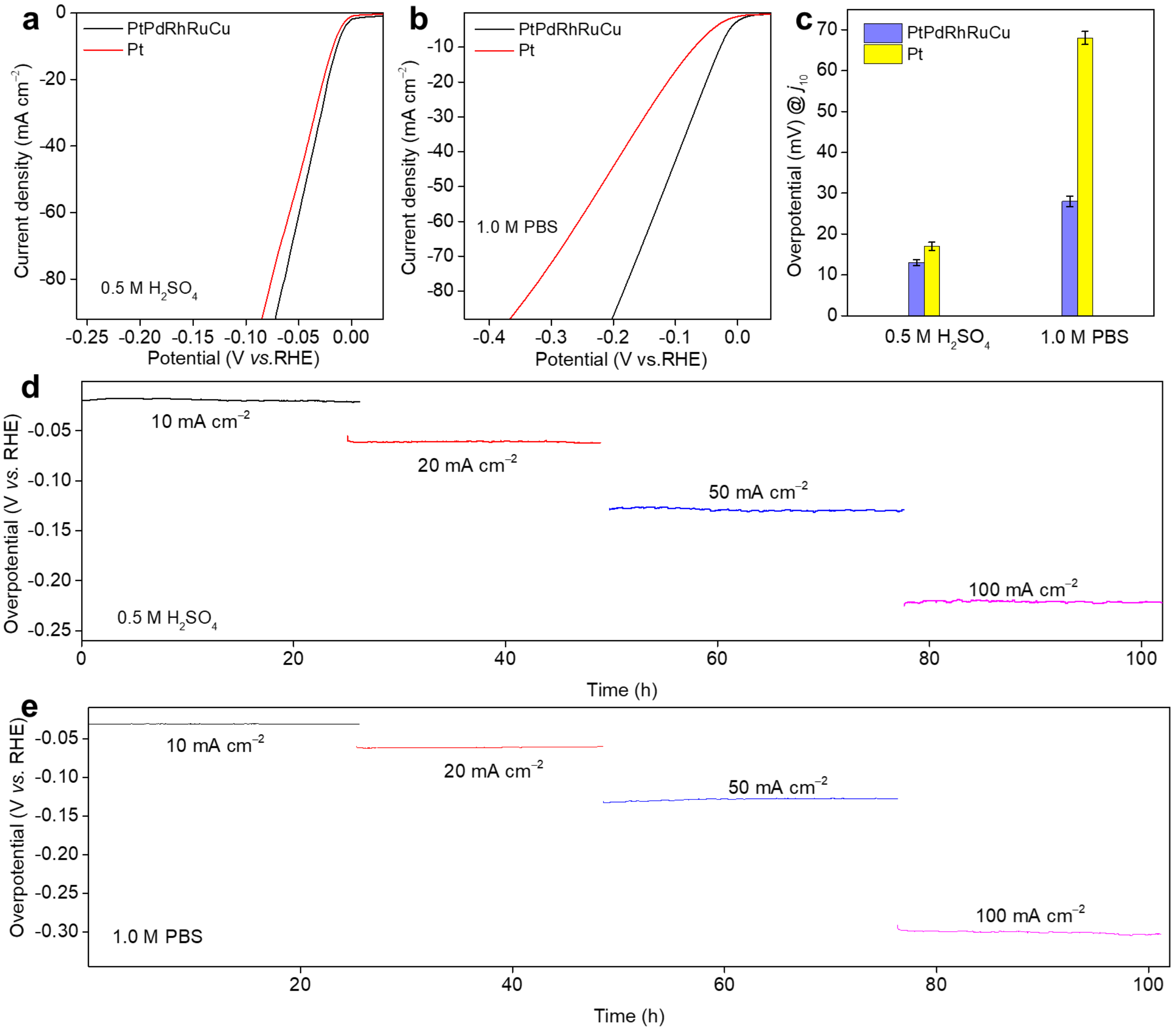

Supplementary Figure 30. Polarization curves of PtPdRhRuCu MMNs and Pt MNs in (a) 0.5 M H_2SO_4 and (b) 1.0 M PBS solutions. (c) Comparison of overpotentials required to achieve a current density of 10 mA cm^{-2}. Error bars obtained from three independent experiments. Long-term chronopotentiometry tests for PtPdRhRuCu MMNs in (d) 0.5 M H_2SO_4 and (e) 1.0 M PBS electrolytes at different current densities. Source data are provided as a Source Data file.

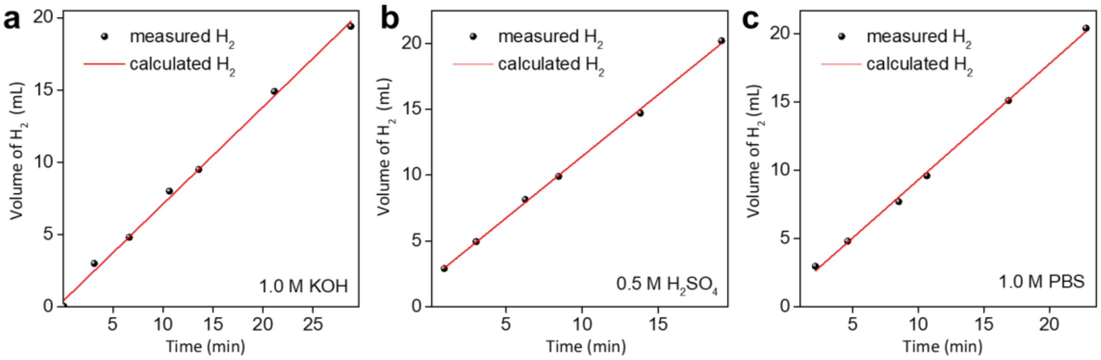

Supplementary Figure 31. The experimentally measured and theoretically calculated amounts of H_2 on PtPdRhRuCu MMNs in different electrolytes. The commercial RuO_2 was used as oxygen evolution reaction catalyst. The applied potential of water splitting for 1.0 M KOH, 0.5 M H_2SO_4, and 1.0 M PBS solution is –2.0, –2.0, and –3.0 V, respectively. Source data are provided as a Source Data file.

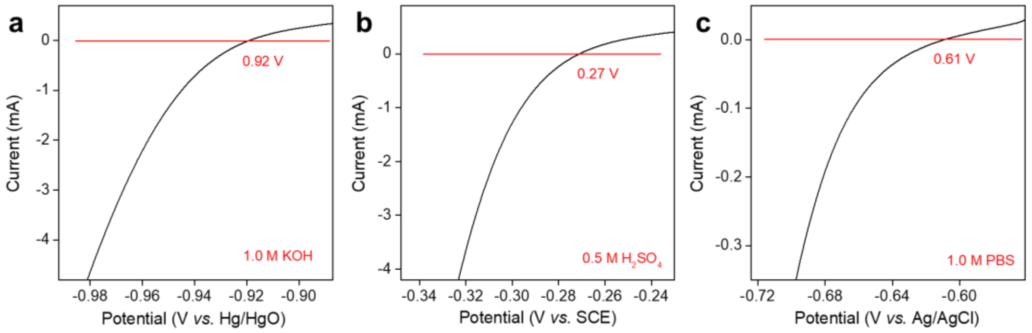

Supplementary Figure 32. Calibration curves of (a) Hg/HgO, (b) SCE, and (c) Ag/AgCl reference in 1.0 M KOH, 0.5 M H_2SO_4, and 1.0 M PBS electrolytes, respectively. The reference electrodes were calibrated with respect to the reversible hydrogen electrode three-electrode system using Pt wires as both the working and counter electrodes.[14] The electrolytes, including 1.0 M KOH, 0.5 M H_2SO_4, and 1.0 M PBS was saturated with high-purity hydrogen for at least 30 min at room temperature and followed CV measurement performed at a scan rate of 1 mV s^{-1}. The potentials at which the currents crossed zero were taken as the thermodynamic potentials. Source data are provided as a Source Data file.

Supplementary Tables

Supplementary Table 1. The ICP–OES analysis of as-prepared and after alkaline HER test of PtPdRhRuCu MMNs.

Sample	Atomic content of the elements (at%)				
	Pt	Pd	Rh	Ru	Cu
As-prepared	23	22	20	13	22
After HER	24	24	19	12	21

Supplementary Table 2. The standard reduction potential of different half reactions for various metal precursors.[15, 16]

Half reaction	E^0 (V *vs.* SHE)
$[PtCl_4]^{2-} + 2e^- \rightarrow Pt + 4Cl^-$	+0.76
$[PdCl_4]^{2-} + 2e^- \rightarrow Pd + 4Cl^-$	+0.59
$[RhCl_6]^{3-} + 3e^- \rightarrow Rh + 6Cl^-$	+0.43
$Ru^{3+} + 3e^- \rightarrow Ru$	+0.39
$Cu^{2+} + 2e^- \rightarrow Cu$	+0.34

Supplementary Table 3. The feeding amounts of various metal precursors and the final compositions of Pt-based MNs.

Sample	Feeding amounts (mL) (40 mM)					Final composition (at%)[a]				
	$[PtCl_4]^{2-}$	$[PdCl_4]^{2-}$	$[RhCl_6]^{3-}$	Ru^{3+}	Cu^{2+}	Pt	Pd	Rh	Ru	Cu
Pt	2.0	-	-	-	-	100	-	-	-	-
PtPd	1.6	0.4	-	-	-	78	22	-	-	-
PtRh	1.6	-	0.4	-	-	83	-	17	-	-
PtRu	1.6	-	-	0.6	-	90	-	-	10	-
PtCu	1.6	-	-	-	0.4	81	-	-	-	19
PtPdRu	1.2	0.4	-	0.6	-	68	21	-	11	-
PtRhRu	1.2	-	0.4	0.6	-	73	-	18	9	-
PtRuCu	-	-	-	0.6	0.4	83	-	-	9	18
PtPdRhRu	0.8	0.4	0.4	0.6	-	46	22	20	12	-
PtRhRuCu	0.8	-	0.4	0.6	0.4	51	-	19	10	20
PtPdRhRuCu	0.4	0.4	0.4	0.6	0.4	23	22	20	11	24

[a]The final compositions of different Pt-based samples were got from SEM-EDS.

Supplementary Table 4. The synthesis conditions for noble-mental-based MNs.

Sample	Preparation parameters					
	Block copolymers	Organic solvent (mL)	Deionized water (mL)	Acid (M, mL)	Temp. (°C)	Time (h)
Pt	PEO_{10500}-*b*-$PMMA_{18000}$	DMF (0.4)	0.5	HCl (6, 0.1)	55	4
Pd[a]	PS_{5000}-*b*-PEO_{2200}	THF (0.2)	0.7	HCl (2, 0.1)	40	4
Rh	PEO_{10500}-*b*-$PMMA_{18000}$	DMF (0.6)	0.4	0	60	12
PtPd	PEO_{10500}-*b*-$PMMA_{18000}$	DMF (0.8)	0.2	HCl (6, 0.4)	60	4
PtM (M = Rh/Ru)	PEO_{10500}-*b*-$PMMA_{18000}$	DMF (0.4)	0	$HClO_4$ (2, 0.7)	50	6
PtRuCu	PEO_{10500}-*b*-$PMMA_{18000}$	DMF (0.4)	0	$HClO_4$ (2, 0.7)	60	6
PtPdRu	PEO_{10500}-*b*-$PMMA_{18000}$	DMF (0.8)	0.2	HCl (6, 0.4)	70	6
PtPdRhRu	PEO_{10500}-*b*-$PMMA_{18000}$	DMF (0.8)	0.2	HCl (6, 0.4)	70	6
PtRhRuCu	PEO_{10500}-*b*-$PMMA_{18000}$	DMF (0.8)	0.2	HCl (6, 0.4)	80	6
PtPdRhRuCu	PEO_{10500}-*b*-$PMMA_{18000}$	DMF (0.8)	0.2	HCl (6, 0.4)	80	6
PtPdRhRuCu-1	PEO_{10000}-*b*-$PMMA_{5500}$	DMF (0.8)	0.2	HCl (6, 0.4)	80	6
PtPdRhRuCu-2	PEO_{10500}-*b*-$PMMA_{22000}$	DMF (0.8)	0.2	HCl (6, 0.4)	80	6
PtPdCuNiCo	PEO_{10500}-*b*-$PMMA_{18000}$	DMF (0.4)	0	$HClO_4$ (2, 0.8)	110	8
PtPdCuNiCoMo	PEO_{10500}-*b*-$PMMA_{18000}$	DMF (0.4)	0	$HClO_4$ (2, 0.8)	110	8

[a] The aqueous H_2PdCl_4 was used for synthesis of Pd MNs while the aqueous Na_2PdCl_4 was used for other PtPd-based MNs.

Supplementary Table 5: The calculation of Löwdin charge[a] analysis for each metal of HEAS, in comparison with the initial valence electron.

Atom	Löwdin charge	Initial valence electrons	Partial charge transfer (|e|)
Pt	10.37	10.0	+ 0.37
Pd	8.51	9.00	– 0.49
Rh	11.19	11.0	+ 0.19
Ru	9.51	10.0	– 0.49
Cu	8.34	8.00	+ 0.34

[a]Löwdin charge was study the charge transfer effects. It depicted the total number of valence electrons for each element. By subtract it to the initial valence electrons of the atom would produce the partial charge of each atom.

Supplementary Table 6. The HER performance of recent reported MMA electrocatalysis at different pH.

Materials	Shape	Preparation method	Electrolyte	Overpotential (mV) @10 mA cm^{-2}	Reference
PtPdRhRuCu	mesoporous nanospheres	soft-templating chemical reduction (80 °C)	1.0 M KOH<br>1.0 M PBS<br>0.5 M H_2SO_4	10<br>28<br>13	This work
NiCoFePtRh	nanoparticles	chemical reduction by SBH and H_2 treatment	0.5 M H_2SO_4	27	13
$Ni_{20}Fe_{20}Mo_{10}Co_{35}Cr_{15}$	bulk	arc-melting 1200 ℃	1.0 M KOH<br>0.5 M H_2SO_4	172<br>107	17
AlNiCuPtPdAu	bulk	melting, fast cooling, and dealloying	0.1.0 M KOH<br>0.5 M H_2SO_4	-<br>-	18
AlNiCoIrMo	bulk	melting pure melts	0.5 M H_2SO_4	18.5	19
PtAuPdRhRu	nanoparticles	chemical reduction by ethylene glycol	1.0 M KOH	-	20
FeCoNiAlTi intermetallic	$L1_2$	arc-melting and dealloying	1.0 M KOH	88.2	21
IrPdPtRhRu	nanoparticles	chemical reduction by triethylene glycol	1.0 M KOH<br>0.05 M H_2SO_4	17<br>33	12
FeCoPdIrPt@GO	nanoparticles	fast moving bed pyrolysis	1.0 M KOH	42	22
CoCrFeMnNiP	bulk	heating with ethylene glycol- 400 ℃	1.0 M KOH	136	23
$Pt_{18}Ni_{26}Fe_{15}Co_{14}Cu_{27}$	nanoparticles	one-pot oil phase synthesis (220 °C)	1.0 M KOH	11	8
CuAlNiMoFe	bulk	arc-melting and dealloying	1.0 M PBS<br>1.0 M KOH	24<br>9.7	24
$Al_{89}Ag_1Au_1Co_1Cu_1Fe_1Ir_1Ni_1Pd_1Pt_1Rh_1Ru_1$	bulk	arc-melting and dealloying	0.5 M H_2SO_4	32	25
PdFeCoNiCu	nanoparticles	one-pot oil phase synthesis (220 °C)	1.0 M KOH	18	26
PdPtCuNiP	metallic glass	large-scalable melt-spinning	1.0 M KOH<br>0.5 M H_2SO_4	32<br>62	27
RuRhPdAgOsIrPtAu	nanoparticles	Heating with triethylene glycol (230 ℃)	0.15 M H_2SO_4	-	11
$Ni_{14}Co_{14}Fe_{14}Mo_6Mn_{52}$	bulk	arc melting and single-roller melt spinning, followed dealloying	1.0 M KOH	14	28
PtRuRhCoNi	nanowire	oleylamine-200 ℃	0.5 M H_2SO_4<br>1.0 M KOH	13<br>15	29
CuCoNiFeMn	bulk	high-energy ball milling	1.0 M KOH<br>0.5 M H_2SO_4	152 @ 70<br>214 @ 20	30

			1.0 M PBS	320 @ 50	
$Co_{0.6}(VMnNiZn)_{0.4}PS_3$	bulk	traditional solid-state reaction with their nominal composition powders	1.0 M KOH	65.9	31
FeCoNiCu	hierarchical porous	physical metallurgy and dealloying strategies	1.0 M KOH	42.2	32
FeCoNiCuPd	bulk	pulsed DC reactive magnetron sputtering	1.0 M KOH	29	33
CoZnCdCuMnS	nanoarrays	hydrothermal	1.0 M KOH	173	34
FeCoNiMnRu	nanoparticles	electrospinning technology and graphitization process.	1.0 M KOH	5	35
CoNiCuMgZn/graphene	nanoparticles	ethylene glycol and thermal treat at 900 ℃	1.0 M KOH<br>0.5 M H_2SO_4	158<br>131	36
IrPdPtRhRu	nanoparticles	continuous-flow reactor	1.0 M $HClO_4$	6	37
FeCoNiCuMnN/CC-400	nanowire/nanosheet	hydrothermal reaction--calcination at 400 °C under Ar/NH_3	1.0 M KOH	184	38
CoCrFeNiAl	bulk	mechanical alloying and spark plasma sintering consolidation	0.5 M H_2SO_4	73	39
$Al_{82}Ni_6Co_3Mn_3Y_3Au_3$	bulk	arc-melting-dealloying	0.5 M H_2SO_4	24	40

Supplementary Table 7: Summary of recently reported Pt-based HER electrocatalysts in different media.

Materials	Shape	Electrolyte	Overpotential (mV) @10 mA cm^{-2}	Reference
PtPdRhRuCu	mesoporous nanospheres	1.0 M KOH	10	This work
		1.0 M PBS	28	
		0.5 M H_2SO_4	13	
$PtNi_5$-0.3	nanoparticles	1.0 M KOH	26.8	[41]
hcp-Pt-Ni	nano-multipods	1.0 M KOH	65	[42]
$PtSn_4$	bulk single crystal	1.0 M KOH	37	[43]
Pt_3Ni_3-NWs	nanowires	1.0 M KOH	40	[44]
Pt-SAs/$MoSe_2$	single atom	1.0 M KOH	29	[45]
PtRu NCs/BP	nanoclusters	1.0 M KOH	22	[46]
PtNi-O/C	nanoparticles	1.0 M KOH	40	[47]
c-PtPd@a-NiB CSHs	hollow nanopolyhedra	1.0 M KOH	31	[48]
Ni-N-C-250/Pt	nanoparticles	1.0 M KOH	83.5	[49]
$PtTe_2$-600 NSs	nanosheets	1.0 M KOH	22	[50]
Pt/np-$Co_{0.85}$Se	single-atom	1.0 M PBS	55	[51]
Pt@CoOx	nanoparticles	1.0 M PBS	82	[52]
BC_3N@Pt	nanoparticles	1.0 M PBS	38.5	[53]
Pt@Cu-0.3	blackberry-shaped nanocrystals	1.0 M PBS	35	[54]
Pt/$PtTe_2$/$NiCoTe_2$	hollow microspheres	1.0 M PBS	36	[55]
$GaPt_3$	nanoparticles	1.0 M PBS	103	[56]
PtAC-MnO_2	cluster	1.0 M PBS	41	[57]
Pt/Ni_3S_2	nanoparticles	1.0 M PBS	109	[58]
Pt-$Co(OH)_2$/CC	nanoparticles	1.0 M PBS	84	[59]
Pd@Pt	nanocube	0.5 M H_2SO_4	15	[60]
$Pt_{3.21}Ni$@Ti_3C_2	ultrathin Nanowires	0.5 M H_2SO_4	18.5	[61]
$Pt_1Cu_{1.03}$	branched nanodendrites	0.5 M H_2SO_4	20	[62]
PtRh	dendritic nanoassemblies	0.5 M H_2SO_4	27	[63]
Pt-RuO_2 HNSs	hollow nanospheres	0.5 M H_2SO_4	26	[64]
Pt_1/OLC	atomically dispersed	0.5 M H_2SO_4	38	[65]
Pt_3Co NPs	nanoparticles	0.5 M H_2SO_4	42	[66]
$La_2Sr_2PtO_{7+\delta}$	bulk	0.5 M H_2SO_4	27	[9]
Pt-WO_3	clusters	0.5 M H_2SO_4	42	[67]
Pt-ACs/CoNC	atomic clusters	0.5 M H_2SO_4	24	[68]
Pt-NPs/CoNC	nanoparticles	0.5 M H_2SO_4	42	[68]

Supplementary References

1. Lim B, *et al.* Pd-Pt Bimetallic nanodendrites with high activity for oxygen reduction. *Science* **324**, 1302-1305 (2009).
2. Slater JC. Atomic radii in crystals. *J. Chem. Phys.* **41**, 3199-3204 (1964).
3. Langford JI, Wilson AJC. Scherrer after sixty years: A survey and some new results in the determination of crystallite size. *J. Appl. Crystallogr.* **11**, 102-113 (1978).
4. Durst J, Siebel A, Simon C, Hasché F, Herranz J, Gasteiger HA. New insights into the electrochemical hydrogen oxidation and evolution reaction mechanism. *Energy Environ. Sci.* **7**, 2255-2260 (2014).
5. Wang P, *et al.* Precise tuning in platinum-nickel/nickel sulfide interface nanowires for synergistic hydrogen evolution catalysis. *Nat. Commun.* **8**, 14580 (2017).
6. Nørskov JK, *et al.* Trends in the exchange current for hydrogen evolution. *J. Electrochem. Soc.* **152**, J23 (2005).
7. Zhan C, *et al.* Subnanometer high-entropy alloy nanowires enable remarkable hydrogen oxidation catalysis. *Nat. Commun.* **12**, 6261 (2021).
8. Li H, *et al.* Fast site-to-site electron transfer of high-entropy alloy nanocatalyst driving redox electrocatalysis. *Nat. Commun.* **11**, 5437 (2020).
9. Dai J, *et al.* Hydrogen spillover in complex oxide multifunctional sites improves acidic hydrogen evolution electrocatalysis. *Nat. Commun.* **13**, 1189 (2022).
10. Yan Y, *et al.* Atomic-level platinum hilling into Ni-vacancies of dual-deficient NiO for boosting electrocatalytic hydrogen evolution. *Adv. Energy Mater.* **12**, 2200434 (2022).
11. Wu D, *et al.* Noble-metal high-entropy-alloy nanoparticles: Atomic-level insight into the electronic structure. *J. Am. Chem. Soc.* **144**, 3365-3369 (2022).
12. Wu D, *et al.* On the electronic structure and hydrogen evolution reaction activity of platinum group metal-based high-entropy-alloy nanoparticles. *Chem. Sci.* **11**, 12731-12736 (2020).
13. Feng G, *et al.* Sub-2 nm Ultrasmall high-entropy alloy nanoparticles for extremely superior electrocatalytic hydrogen evolution. *J. Am. Chem. Soc.* **143**, 17117-17127 (2021).
14. Yang Y, *et al.* Hierarchical Nanoassembly of MoS_2/Co_9S_8/Ni_3S_2/Ni as a highly efficient electrocatalyst for overall water splitting in a wide pH range. *J. Am. Chem. Soc.* **141**, 10417-10430 (2019).
15. Jiang B, Li C, Malgras V, Yamauchi Y. Synthesis of ternary PtPdCu spheres with three-dimensional nanoporous architectures toward superior electrocatalysts. *J. Mater. Chem. A* **3**, 18053-18058 (2015).
16. Jiang B, *et al.* Mesoporous trimetallic PtPdRu spheres as superior electrocatalysts. *Chem. Eur. J.* **22**, 7174-7178 (2016).
17. Zhang G, *et al.* High entropy alloy as a highly active and stable electrocatalyst for hydrogen evolution reaction. *Electrochim. Acta* **279**, 19-23 (2018)
18. Qiu H-J, *et al.* Nanoporous high-entropy alloys for highly stable and efficient catalysts. *J. Mater. Chem. A* **7**, 6499-6506 (2019).
19. Jin Z, *et al.* Nanoporous Al-Ni-Co-Ir-Mo high-entropy alloy for record-high water splitting activity in acidic environments. *Small* **15**, 1904180 (2019).

20. Liu M, Zhang Z, Okejiri F, Yang S, Zhou S, Dai S. Entropy-maximized synthesis of multimetallic nanoparticle catalysts via a ultrasonication-assisted wet chemistry method under ambient conditions. *Adv. Mater. Interfaces* **6**, 1900015 (2019).
21. Jia Z*, et al.* A novel multinary intermetallic as an active electrocatalyst for hydrogen evolution. *Adv. Mater.* **32**, 2000385 (2020).
22. Gao S*, et al.* Synthesis of high-entropy alloy nanoparticles on supports by the fast moving bed pyrolysis. *Nat. Commun.* **11**, 2016 (2020).
23. Zhao X, Xue Z, Chen W, Wang Y, Mu T. Eutectic synthesis of high-entropy metal phosphides for electrocatalytic water splitting. *ChemSusChem* **13**, 2038-2042 (2020).
24. Yao R-Q*, et al.* Nanoporous surface high-entropy alloys as highly efficient multisite electrocatalysts for nonacidic hydrogen evolution reaction. *Adv. Funct. Mater.* **31**, 2009613 (2021).
25. Cai Z-X*, et al.* Nanoporous ultra-high-entropy alloys containing fourteen elements for water splitting electrocatalysis. *Chem. Sci.* **12**, 11306-11315 (2021).
26. Zhang D*, et al.* The facile oil-phase synthesis of a multi-site synergistic high-entropy alloy to promote the alkaline hydrogen evolution reaction. *J. Mater. Chem. A* **9**, 889-893 (2021).
27. Jia Z*, et al.* A Self-supported high-entropy metallic glass with a nanosponge architecture for efficient hydrogen evolution under alkaline and acidic conditions. *Adv. Funct. Mater.* **31**, 2101586 (2021).
28. Liu H*, et al.* A freestanding nanoporous NiCoFeMoMn high-entropy alloy as an efficient electrocatalyst for rapid water splitting. *Chem. Eng. J.* **435**, 134898 (2022).
29. Li H*, et al.* The self-complementary effect through strong orbital coupling in ultrathin high-entropy alloy nanowires boosting pH-universal multifunctional electrocatalysis. *Appl. Catal. B* **312**, 121431 (2022).
30. Sivanantham A, Lee H, Hwang SW, Ahn B, Cho IS. Preparation, electrical and electrochemical characterizations of CuCoNiFeMn high-entropy-alloy for overall water splitting at neutral-pH. *J. Mater. Chem. A* **9**, 16841-16851 (2021).
31. Wang R*, et al.* Two-dimensional high-entropy metal phosphorus trichalcogenides for Enhanced hydrogen evolution reaction. *ACS Nano* **16**, 3593-3603 (2022).
32. Li R, Liu X, Liu W, Li Z, Chan KC, Lu Z. Design of hierarchical porosity via manipulating chemical and microstructural complexities in high-entropy alloys for efficient water electrolysis. *Adv. Sci.* **9**, 2105808 (2022).
33. Wang S*, et al.* Efficient FeCoNiCuPd thin-film electrocatalyst for alkaline oxygen and hydrogen evolution reactions. *Appl. Catal. B* **313**, 121472 (2022).
34. Lei Y*, et al.* Carbon-supported high-entropy Co-Zn-Cd-Cu-Mn sulfide nanoarrays promise high-performance overall water splitting. *Nano Research* **15**, 6054-6061 (2022).
35. Hao J*, et al.* Unraveling the electronegativity-dominated intermediate adsorption on high-entropy alloy electrocatalysts. *Nat. Commun.* **13**, 2662 (2022).
36. Feng D, Dong Y, Nie P, Zhang L, Qiao Z-A. CoNiCuMgZn high entropy alloy nanoparticles embedded onto graphene sheets via anchoring and alloying strategy as efficient electrocatalysts for hydrogen evolution reaction. *Chem. Eng. J.* **430**, 132883 (2022).

37. Minamihara H, *et al.* Continuous-flow reactor synthesis for homogeneous 1 nm-sized extremely small high-entropy alloy nanoparticles. *J. Am. Chem. Soc.* **144**, 11525-11529 (2022).
38. Liu C, Zhu H, Lu S, Duan F, Du M. High entropy alloy nitrides with integrated nanowire/nanosheet architecture for efficient alkaline hydrogen evolution reactions. *New J. Chem.* **45**, 22255-22260 (2021).
39. Ma P, *et al.* Self- supported high-entropy alloy electrocatalyst for highly efficient H2 evolution in acid condition. *J. Materiomics* **6**, 736-742 (2020).
40. Liu X, *et al.* Advanced catalyst for hydrogen evolution reaction by dealloying Al-based nanocrystalline alloys. *J. Alloys Compd.* **880**, 160548 (2021).
41. Zhang C, *et al.* H_2 In situ inducing strategy on Pt surface segregation over low Pt Doped $PtNi_5$ nanoalloy with superhigh alkaline HER activity. *Adv. Funct. Mater.* **31**, 2008298 (2021).
42. Cao Z, *et al.* Platinum-nickel alloy excavated nano-multipods with hexagonal close-packed structure and superior activity towards hydrogen evolution reaction. *Nat. Commun.* **8**, 15131 (2017).
43. Li G, *et al.* Dirac nodal arc semimetal $PtSn_4$: An ideal platform for understanding surface properties and catalysis for hydrogen evolution. *Angew. Chem. Int. Ed.* **58**, 13107-13112 (2019).
44. Wang P, Jiang K, Wang G, Yao J, Huang X. Phase and interface engineering of platinum–nickel nanowires for efficient electrochemical hydrogen evolution. *Angew. Chem. Int. Ed.* **55**, 12859-12863 (2016).
45. Shi Y, *et al.* Electronic metal–support interaction modulates single-atom platinum catalysis for hydrogen evolution reaction. *Nat. Commun.* **12**, 3021 (2021).
46. Li Y, *et al.* Hybrids of PtRu nanoclusters and black phosphorus nanosheets for highly efficient alkaline hydrogen evolution reaction. *ACS Catal.* **9**, 10870-10875 (2019).
47. Zhao Z, *et al.* Surface-engineered PtNi-O nanostructure with record-high performance for electrocatalytic hydrogen evolution reaction. *J. Am. Chem. Soc.* **140**, 9046-9050 (2018).
48. Deng K, *et al.* Crystalline core–amorphous shell heterostructures: epitaxial assembly of NiB nanosheets onto PtPd mesoporous hollow nanopolyhedra for enhanced hydrogen evolution electrocatalysis. *J. Mater. Chem. A* **8**, 8927-8933 (2020).
49. Li P, *et al.* Nickel single atom-decorated carbon nanosheets as multifunctional electrocatalyst supports toward efficient alkaline hydrogen evolution. *Nano Energy* **83**, 105850 (2021).
50. Li X, *et al.* Ordered clustering of single atomic Te vacancies in atomically thin $PtTe_2$ promotes hydrogen evolution catalysis. *Nat. Commun.* **12**, 2351 (2021).
51. Jiang K, *et al.* Single platinum atoms embedded in nanoporous cobalt selenide as electrocatalyst for accelerating hydrogen evolution reaction. *Nat. Commun.* **10**, 1743 (2019).
52. Zhai L, *et al.* Modulating built-in electric field via variable oxygen affinity for robust Hydrogen evolution reaction in neutral media. *Angew. Chem. Int. Ed.* **61**, e202116057 (2022).
53. Zhao X, *et al.* Supramolecular nanosheet evolution into BC_3N matrix improves the hydrogen evolution reaction activity in the pH universality of highly dispersed Pt nanoparticles. *J. Mater. Chem. A* **9**, 16427-16435 (2021).
54. Tan Y, *et al.* Facile fabrication of robust hydrogen evolution electrodes under high current densities via Pt@Cu interactions. *Adv. Funct. Mater.* **31**, 2105579 (2021).

55. Yi M*, et al.* Multicomponent $Pt/PtTe_2/NiCoTe_2$ embedded in ternary heteroatoms-doped carbon for efficient and pH-universal hydrogen evolution reaction. *J. Alloys Compd.* **884**, 161042 (2021).
56. Lim S-C, Chan C-Y, Chen K-T, Tuan H-Y. Synthesis of popcorn-shaped gallium-platinum ($GaPt_3$) nanoparticles as highly efficient and stable electrocatalysts for hydrogen evolution reaction. *Electrochim. Acta* **297**, 288-296 (2019).
57. Wei J-X, Cao M-Z, Xiao K, Guo X-P, Ye S-Y, Liu Z-Q. In situ confining Pt clusters in ultrathin MnO_2 nanosheets for highly efficient hydrogen evolution reaction. *Small Struct.* **2**, 2100047 (2021).
58. Xing Z, Wang D, Meng T, Yang X. Superb hydrogen evolution by a Pt nanoparticle-decorated Ni_3S_2 microrod array. *ACS Appl. Mater. Interfaces* **12**, 39163-39169 (2020).
59. Xing Z, Han C, Wang D, Li Q, Yang X. Ultrafine Pt nanoparticle-decorated $Co(OH)_2$ nanosheet arrays with enhanced catalytic activity toward hydrogen evolution. *ACS Catal.* **7**, 7131-7135 (2017).
60. Bai S*, et al.* Surface polarization matters: Enhancing the hydrogen-evolution reaction by shrinking Pt shells in Pt–Pd–graphene stack structures. *Angew. Chem. Int. Ed.* **53**, 12120-12124 (2014).
61. Jiang Y*, et al.* Coupling PtNi ultrathin nanowires with MXenes for boosting electrocatalytic hydrogen evolution in both acidic and alkaline solutions. *Small* **15**, 1805474 (2019).
62. Du Y*, et al.* Facile air oxidative induced dealloying of hierarchical branched PtCu nanodendrites with enhanced activity for hydrogen evolution. *Appl. Catal. A* **557**, 72-78 (2018).
63. Han Z*, et al.* Platinum-rhodium alloyed dendritic nanoassemblies: An all-pH efficient and stable electrocatalyst for hydrogen evolution reaction. *Int. J. Hydrogen Energy* **45**, 6110-6119 (2020).
64. Wang J*, et al.* Single-site Pt-doped RuO_2 hollow nanospheres with interstitial C for high-performance acidic overall water splitting. *Sci. Adv.* **8**, eabl9271 (2022).
65. Liu D*, et al.* Atomically dispersed platinum supported on curved carbon supports for efficient electrocatalytic hydrogen evolution. *Nat. Energy* **4**, 512-518 (2019).
66. Zhang SL, Lu XF, Wu Z-P, Luan D, Lou XW. Engineering platinum–cobalt nano-alloys in porous nitrogen-doped carbon nanotubes for highly efficient electrocatalytic hydrogen evolution. *Angew. Chem. Int. Ed.* **60**, 19068-19073 (2021).
67. Xie C*, et al.* In-situ phase transition of WO_3 boosting electron and hydrogen transfer for enhancing hydrogen evolution on Pt. *Nano Energy* **71**, 104653 (2020).
68. Zhao Y*, et al.* Modulating Pt-O-Pt atomic clusters with isolated cobalt atoms for enhanced hydrogen evolution catalysis. *Nat. Commun.* **13**, 2430 (2022).
